# Precise control of transmembrane current via regulating bionic lipid membrane composition

**DOI:** 10.1126/sciadv.adq0118

**Published:** 2024-08-30

**Authors:** Zhiwei Shang, Jing Zhao, Mengyu Yang, Yuling Xiao, Wenjing Chu, Shijun Xu, Xiaojin Zhang, Xiaoqing Yi, Meihua Lin, Fan Xia

**Affiliations:** ^1^ State Key Laboratory of Biogeology and Environmental Geology, Engineering Research Center of Nano-Geomaterials of Ministry of Education, Faculty of Materials Science and Chemistry, China University of Geosciences, Wuhan 430074, China.; ^2^ Key Laboratory of Prevention and Treatment of Cardiovascular and Cerebrovascular Diseases, Ministry of Education, Gannan Medical University, Ganzhou 341000, China.

## Abstract

The transport of ions through biological ion channels is regulated not only by their structural characteristics but also by the composition of the phospholipid membrane, which serves as a carrier for nanochannels. Inspired by the modulation of ion currents by lipid membrane composition, exemplified by the activation of the K^+^ channel of *Streptomyces* A by anionic lipids, we present a biomimetic nanochannel system based on combining DNA nanotechnology with two-dimensional graphene oxide (GO) nanosheets. By designing multibranched DNA nanowires, we assemble programmable DNA scaffold networks (DSNs) on the GO surface to precisely control membrane composition. Modulating the DSN layers from one to five enhances DNA composition, yielding a maximum 12-fold enhancement in ion current, primarily due to charge effects. Incorporating DNAzymes facilitates reversible modulation of membrane composition, enabling cyclic conversion of ion current. This approach offers a pathway for creating devices with highly efficient, tunable ion transport, applicable in diverse fields like mass transport, environmental protection, biomimetic channels, and biosensors.

## Teaser

Transmembrane ion current is accurately controlled by programmatically modulating the composition of the bionic lipid membrane.

## INTRODUCTION

In nature, the transport of ions across the cell membrane is essential for many fundamental biological processes (*1*, *2*). Ion transport occurs via transmembrane protein pores, which is affected not only by the structural characteristics of protein pores but also the phospholipid membrane compositions (*3*, *4*). The phospholipid membrane acts as a scaffold for protein pores, and its composition, such as the content of phospholipids, proteins, peptides, polysaccharides, and cholesterol, plays an important role in regulating ion transport (*5*–*9*). In particular, biological ion channels can be regulated by phospholipid membrane composition to establish a connection between the metabolic state of a cell and its electrical activity (*10*, *11*). Membrane bulk properties such as hydrophobic thickness, lipid asymmetry, local curvature, elasticity, or electrostatics actively modulate protein function (*12*–*15*). In addition, certain individual lipids are important messengers in biological processes (*16*, *17*). For instance, potassium channel KcsA (K^+^ channel of *Streptomyces* A) derived from *Streptomyces lividans* is sensitive to an anionic lipid, 1-palmitoyl-2-oleoyl phosphatidylglycerol (POPG), which is present in the phospholipid membrane (*18*). When POPG is introduced into the membrane, its ability to bind to the channel and modify its conformation enhances the opening probability of KcsA. Simultaneously, the inclusion of negatively charged lipids in the membrane elevates the concentration of K^+^ ions near the membrane surface, resulting in a notable increase in the K^+^ concentration proximal to the channel pore. This elevated K^+^ concentration near the channel pore significantly augments ion conductivity. Therefore, with the increase in POPG concentration, the ion current of KcsA gradually rises.

Motivated by transmembrane protein pores in living cells, many single protein pores, such as α-hemolysin, have been inserted into the lipid bilayer to construct an artificial nanofluidic system for many applications (*19*–*22*). The compositions of the lipid bilayer are simpler than that of the cell membrane, so it is more convenient to investigate the effect of lipid membrane composition on ion transport. For example, the effect of lipid compositions (including different kinds of anionic phospholipid and phosphatidylethanolamine) on the *Erwinia* ligand-gated ion channel function has been assessed by reconstituting the channel in giant liposomes (*23*). The successful lipid modulation of ion channel function showcases a promising avenue for exploring the quantitative relationship between the activity of biological ion channels and cell membrane composition as well as for developing highly sensitive sensors (*24*, *25*). Nevertheless, the heterogeneous structure of phospholipid membranes is well confirmed, and these lateral heterogeneities are dynamic, assembling or disappearing in response to various parameters (*12*). This dynamic nature makes it challenging to precisely control the distribution of functional molecules on the membrane. Moreover, the stability and strength of phospholipid membranes are relatively weak, which affects the further application of artificial protein ion channels regulated by phospholipid membrane compositions (*26*). With the development of materials and nanofabrication technology, solid-state ion nanochannels with robust mechanical and adjustable chemical properties have sprung up, which have been widely applied for DNA sequencing, sensing, separation, and energy conversion (*27*–*32*). An existing report has indicated that the modification of a two-dimensional (2D) nanofluidic graphene oxide (GO) surface with functional molecules of varying charges can regulate ion transport within the channels (*33*). Using polyelectrolytes with distinct charge densities as functional molecules endows controllable surface charges to the GO layers. The interlayer ion transport is predominantly governed by the surface charge of the functional molecules, which indicates that ion transport can be regulated by modulating the composition of the biomimetic membrane. However, it is challenging to achieve control of functional molecular density at a quantitative or semiquantitative level with currently available methods, which are largely empirical and often rely on parameter optimization (*34*, *35*). This issue greatly restricts the precise modulation of ion transport through solid-state nanochannels.

Recently, DNA nanotechnology with precise programmability and high predictability has been rapidly developed (*36*–*43*). Benefiting from the specific base-pair recognition, DNA nanotechnology has shown excellent results in the precise and controllable assembly of DNA nanostructures of various sizes and geometric shapes (*44*–*50*). Among the DNA nanostructures, long double-stranded DNA nanowires prepared by a hybridization chain reaction or supersandwich approach have attracted increased attention (*51*, *52*). With rational design, the 1D DNA nanowires can be reengineered to multibranched nanowires to act as building blocks to assemble hierarchical nanostructures, and the length of multibranched DNA nanowires can be tuned by the proportion of the reactant DNA (*53*). The layer-by-layer assembly of multibranched DNA nanowires can be used to construct DNA scaffold networks (DSNs) (*54*). Thus, the size of DSNs can be finely tuned by the varying the number of layers of multibranched DNA nanowires, which can be used to precisely engineer the membrane surface, showing potential to accurately regulate the membrane compositions.

**Fig. 1.**
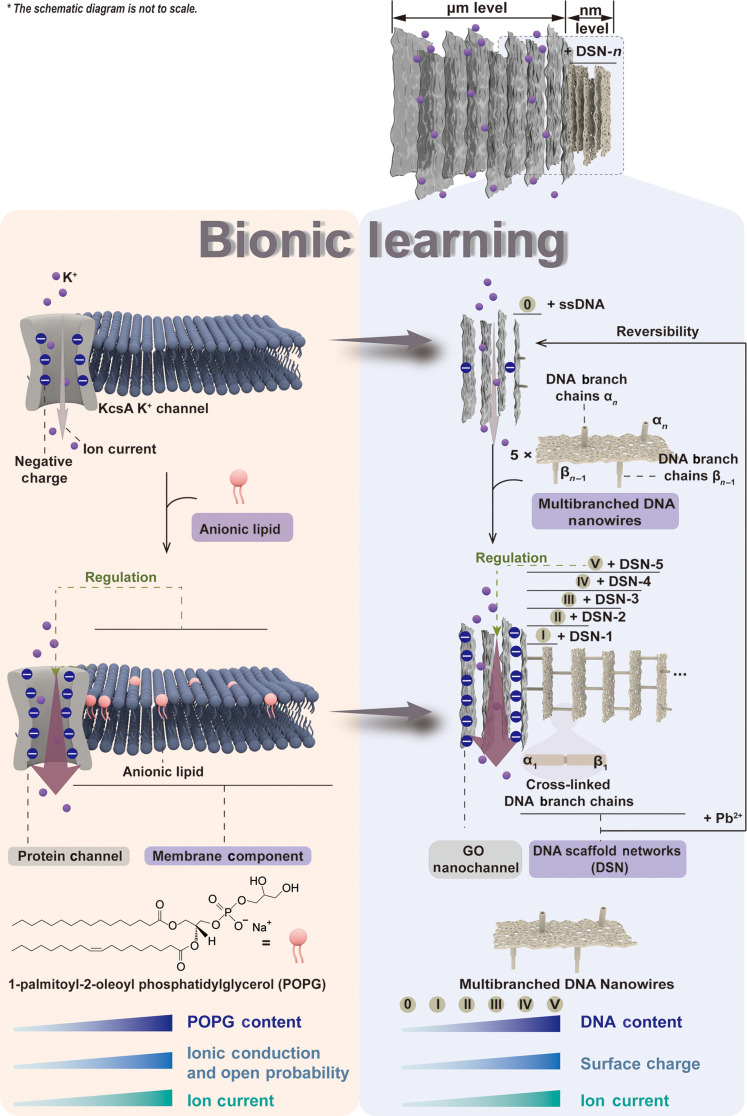
Schematic representation of the control of transmembrane current via modulating bionic lipids. (Left) In biological transmembrane pore proteins, ion/molecular transport can be modulated by the exposure of charged residues, which is controlled by the phospholipid membrane composition, such as anionic lipid POPG. (Right) Inspired by this, a biomimetic nanochannel system is constructed by self-assembling tunable DSNs on the surface of GO nanochannels. DSNs are assembled by programmable layers of multibranched DNA nanowires to accurately regulate the membrane composition. In this process, we investigate the effect of DSNs on the regulation of ion transport. Moreover, when incorporating Pb^2+^-specific DNAzyme into DSNs, the DSN-5@GO membrane can be reversely switched to the ssDNA@GO membrane with the aid of Pb^2+^ ions, leading to the detachment of DSNs from the GO surface and thereby reversely regulating ion transport. The ssDNA@GO membrane is capable of reassembling DSN nanostructures, thus regenerating the DSN-5@GO membrane. *, the schematic diagram is not to scale.

Herein, we combine DNA nanotechnology with 2D nanosheets to construct a biomimetic nanochannel system for the precise modulation of ion transport by precisely regulating the membrane composition (Fig. 1 and fig. S1). GO nanosheets were chosen as nanochannels and supporting membranes due to their intrinsic tunable subnanometer interlayer spacing, rich surface chemistry, and excellent compatibility with DNA. As key functional modules, multibranched DNA nanowires hybridize with single-stranded DNA (ssDNA) adsorbed on the surface of the GO membrane to assemble the intricate architecture of DSNs. The membrane composition of DSNs can be accurately modulated by gradually increasing the number of assembled layers of multibranched DNA nanowires (denoted as “*n*” in DSN-*n*), resulting in the precise regulation of ion transport. With each additional layer of DSNs, a stepwise increase in current ranging from 0.20 to 0.65 μA is observed. The experimental and theoretical results demonstrate that the ion current increases with the increased layers in DSNs, indicating that the ion transport of the developed biomimetic membranes can be regulated by the increased contents of DNA of the membrane due to the dominant role of the charge effect. Meanwhile, owing to the high programmability and spatial addressability of DNA nanostructures, various functional molecules and nanomaterials, including proteins, gold nanoparticles (AuNPs), and DNAzymes, have been precisely integrated into DSNs to regulate ion transport, diversifying the regulation of membrane compositions. By integrating Pb^2+^-specific DNAzyme into DSNs, the DSNs anchored on the surface of the GO membrane can be released upon exposure to Pb^2+^, leading to a reduction in ion current. Through replenishing DSNs containing the cleaved substrates, the reassembly progress of DSNs onto the GO membrane can be achieved. The membrane composition can be reversibly regulated in multiple cycles through the assembly-disassembly-reassembly progress of DSNs, prompting the reversible conversion of ion current. The DSN-based biomimetic membrane is characterized by meticulously engineered structure and ion transport regulation capabilities, which serves as the cornerstone for developing devices capable of achieving highly efficient ion transport tailored to specific requirements.

## RESULTS

### Design and characterization of tunable DSNs

**Fig. 2.**
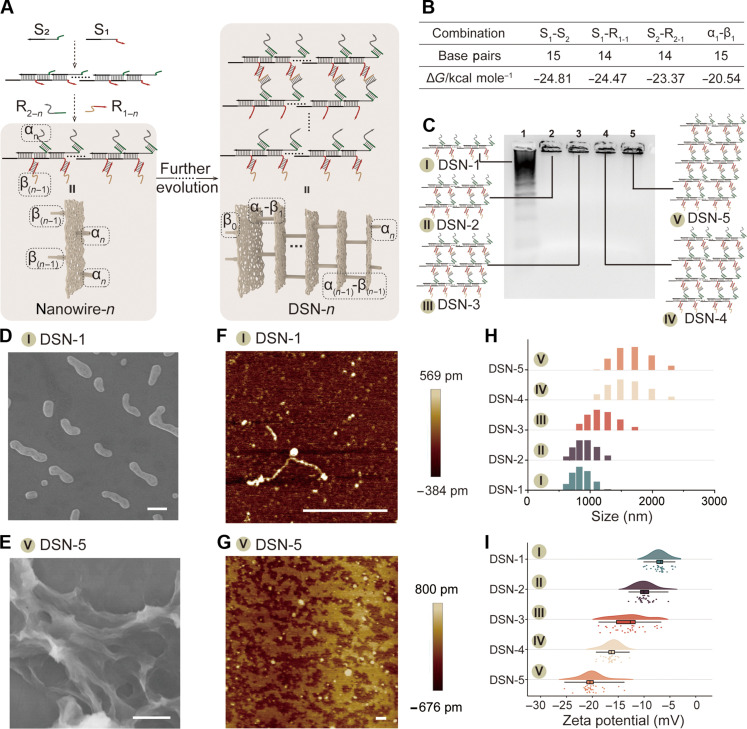
Construction and characterization of tunable DSNs. (**A**) Schematic illustration of the synthesis program for DSNs. (**B**) Theoretical calculation data of DSNs. (**C**) AGE assay of tunable DSN-*n*. From lanes 1 to 5: DSN-1, DSN-2, DSN-3, DSN-4, and DSN-5. SEM images of (**D**) DSN-1 and (**E**) DSN-5 structures. AFM images of (**F**) DSN-1 and (**G**) DSN-5 structures. Scale bars, 500 nm. (**H**) Hydrodynamic diameter distribution of DSN-*n* networks in the buffer solution with pH 7.4. (**I**) Scatter dot plots showing the zeta potential for DSN-*n* networks measured at pH 7.4 (*N* = 30 repeats).

As a test bed for construction of DSNs with different sizes, we designed five types of multibranched DNA nanowires: nanowire-1, nanowire-2, nanowire-3, nanowire-4, and nanowire-5, where each contains the same main backbone but different branched sequences [named as α*_n_* and β_(*n*−1)_, respectively] (Fig. 2A and fig. S2). With rational design, the branched sequences within neighboring multibranched DNA nanowires could undergo hybridization, such as α*_n_* in nanowire-*n* hybridizing with β*_n_* in nanowire-(*n*+1), facilitating the construction of DSNs. The size of DSNs could be precisely controlled by the number of layers in the multibranched DNA nanowires. For instance, when comprising *n* layers of multibranched DNA nanowires, the resulting structure was termed as DSN-*n* (Fig. 1A). The self-assembly process of DSNs is controlled by the sequences of α*_n_* and β*_n_*. With the assistance of NUPACK (www.nupack.org), we designed the sequences and calculated the standard free energy of DNA hybridization. The calculations were in a range between −20 and −25 kcal mol^−1^ (Fig. 2B and fig. S3), indicating the stability of the hybridized structures. Figure S4 confirmed the successful synthesis of the multibranched DNA nanowire structure (from nanowire-1 to nanowire-5). Agarose gel electrophoresis (AGE) analysis demonstrated the successful assembly of these DSN structures. Furthermore, it was observed that the DSN remained localized within the loading hole when the number of layers in the multibranched DNA nanowires exceeded one (Fig. 2C). We conducted further analysis of DSN-1 and DSN-5 using scanning electron microscopy (SEM) and atomic force microscopy (AFM). As shown in Fig. 2 (D and F) and fig. S5, we observed 1D nanowires within a length range of ~330 to 1160 nm, demonstrating the successful assembly of DSN-1. In contrast, the morphology of DSN-5 depicted a network structure (Fig. 2, E and G), suggesting the layer-by-layer assembly of nanowires in the formation of DSN-5. The hydrodynamic diameter of DSN-*n*, as measured by dynamic light scattering (DLS), exhibited a direct correlation with the number of layers of multibranched DNA nanowires, as illustrated in Fig. 2H. Specifically, the hydrodynamic diameter of DSN-1 fell within the range of ~600 to 1200 nm, slightly larger than its dimensions in the dry state due to DNA hydration. Furthermore, in line with the DLS findings, there was a consistent and monotonic increase observed in the zeta potential with the expanding size of the DSN, as depicted in Fig. 2I. These findings collectively indicated the efficient and programmable assembly of DSN structures.

### Construction and characterization of the ssDNA@GO membrane

To create a biomimetic membrane with precisely adjustable composition, the combination of DSNs with the GO membrane is crucial. Hence, we designed an ssDNA with polyadenine sequences and recognition sequences for anchoring on the GO membrane (figs. S6 to S19) and hybridization with β_0_ in DSNs, respectively. The ssDNA could adsorb onto the surface of the GO membrane to fabricate the ssDNA@GO membrane through hydrophobic and π-π stacking interaction between polyadenine sequences and GO (*55*, *56*). Compared to covalent modification, using a polyadenine sequence to modify DNA on the surface of GO has a cost advantage. The cost of DNA synthesis when using the polyadenine sequence can be reduced by at least 90% compared to modifying chemical groups (*57*). Furthermore, the length of the polyadenine sequence can be adjusted to systematically control the lateral spacing and surface density of the recognition sequences on the GO surface (*58*). We used spectrofluorometry (fig. S20), confocal microscopy (fig. S21), and x-ray photoelectron spectroscopy (figs. S22 and S23) to confirm the modification of ssDNA on the surface of the GO membrane. Subsequently, we investigated the effect of ssDNA on ion transport of the GO membrane using a 10 μM KCl solution in a two-electrode system with symmetric Ag/AgCl electrodes (figs. S18 and S24). As depicted in fig. S25, the ion current increased after the assembly of ssDNA due to the negatively charged DNA. The stability of ssDNA@GO for ion transport was also investigated. As illustrated in fig. S26, the ion currents obtained from 30 cycles remained nearly consistent with each other, indicating the excellent stability of the ssDNA@GO membrane. Therefore, we have successfully fabricated the ssDNA@GO membrane for the further construction of a biomimetic nanochannel system.

### Tunable membrane compositions regulate ion transport

**Fig. 3.**
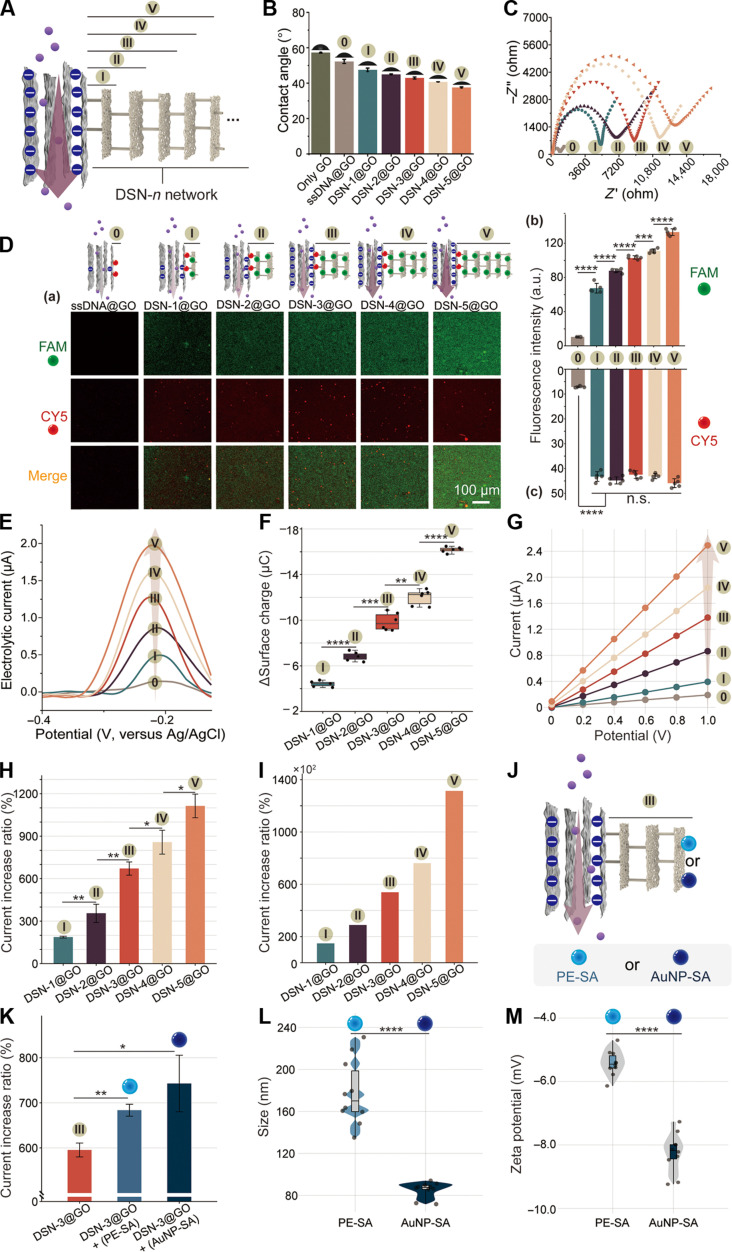
Design and characterization the role of the bionic membrane composition on regulating ion transport. (**A**) Schematic illustration of ion current regulation by the tunable DSN-*n* network. (**B**) Contact angle for the GO, ssDNA@GO and DSN-*n*@GO membrane. The data represent the means ± SD (error bars) (*N* = 3). (**C**) EIS for ssDNA and DSN-*n* networks on the gold electrode surface. (**D**) (a) Confocal images of the DSN-*n*@GO membrane (CY5-tagged ssDNA and FAM-tagged multibranched DNA nanowires). Scale bar, 100 μm. Statistical analysis of the fluorescence intensity of (b) FAM and (c) CY5 based on the corresponding data in (a). The data represent the means ± SD (*N* = 5). ***P < 0.001; ****P < 0.0001; n.s., not significant by *t* test. (**E**) The DSN assembly progress was investigated by using electrochemical SWV. (**F**) Surface charge variation of the DSN-*n*@GO membrane (*N* = 6). **P < 0.01; ***P < 0.001; ****P < 0.0001. (**G**) Typical *I*-*V* curves of the ssDNA@GO and DSN-*n*@GO membrane. (**H**) Ion current increase ratio [(*I* − *I*_0_)/*I*_0_ × 100% at +1.0 V] after the assembly of DSN-*n*. The data represent the means ± SD (*N* = 3). *P < 0.05; **P < 0.01. (**I**) Ion current increase ratio after the assembly of DSN-*n* was calculated by numerical simulation. The variation regularity is consistent with the experimental results. (**J**) Schematic representation illustrating the versatility of the membrane composition modulation via integrating PE-SA or AuNP-SA into DSNs. (**K**) Ion current increase ratio after the assembly of DSN-3 with PE-SA or AuNP-SA. The data represent the means ± SD (*N* = 3). *P < 0.05; **P < 0.01 by *t* test. Scatter dot plots showing (**L**) the hydrodynamic diameter and (**M**) the zeta potential of PE-SA and AuNP-SA (*N* = 10 repeats). The samples were tested in the hybridization buffer [10 mM PB, 20 mM Mg^2+^, and 1 M NaCl (pH 7.4)]. ****P < 0.0001.

After the successful construction of DSN-*n* networks in a buffer solution and the successful preparation of the ssDNA@GO membrane, we proceeded to fabricate a biomimetic nanochannel system with tunable membrane compositions to precisely modulate ion transport. As depicted in fig. S27, the ssDNA on the surface of the GO membrane was capable of hybridizing with β_0_ in nanowire-1, resulting in the construction of the DSN-1@GO membrane. Subsequently, using a layer-by-layer assembly process similar to that in the buffer solution, we were able to precisely fabricate the DSN-*n*@GO membrane (Fig. 3A), wherein the size of DSNs became larger as *n* increased (fig. S28). The contact angles of the DSN-*n*@GO membrane exhibited a decreasing trend with the increasing size of DSNs, suggesting enhanced hydrophilicity of the membrane with the increased compositions of DNA (Fig. 3B). We then used a gold electrode for the assembly of DSNs following a similar process as on the GO membrane (fig. S29). Thus, electrochemical impedance spectroscopy (EIS) was used to validate the fabrication of DSNs through the stepwise hybridization of various multibranched DNA nanowires on the gold electrode surface (*59*). The semicircle diameter observed in the Nyquist plot demonstrated a monotonic increase with the rise in the DSN layer (Fig. 3C), attributed to the heightened negative surface charge of DNA hindering the diffusion of ferricyanide. This result indicated that the DSN could be successfully assembled in a controlled manner on the surface. To further confirm the localization of the assembly of DSNs on the surface of the GO membrane, we monitored the self-assembly process by tagging the ssDNA and multibranched DNA nanowires with Cyanine5 (CY5) and 6-carboxyfluorescein (FAM), respectively. Upon gradually introducing DNA multibranched nanowires to the ssDNA@GO membrane, we observed fluorescence emitting from FAM and CY5 on the surface of the GO membrane [Fig. 3D(a)]. As illustrated in Fig. 3D(b), the fluorescence intensity of FAM exhibited an increase ~2.2 times during the assembly of DSN-*n*@GO from DSN-1 to DSN-5, indicating the programmable and successful modulation of DSNs on the GO membrane, whereas CY5 fluorescence was initially quenched at the state 0 due to ssDNA adsorption on the GO membrane, and subsequently, the intensity notably increased owing to the hybridization of β_0_ in nanowire-1 with recognition sequences on the GO membrane, which caused the fluorophores to move away from the GO surface. However, the fluorescence intensity of CY5 remained relatively stable upon further assembly of more layers of DSNs [Fig. 3D(c)], indicating strong binding of the anchoring sequences with the GO surface to fabricate the DSN-*n*@GO membrane. Furthermore, square-wave voltammetry (SWV) was used to investigate the progression of DSN assembly using methylene blue (MB), which binds to the DNA structure via electrostatic and π-stacking interactions, generating an electrochemical signal (*60*, *61*). As depicted in Fig. 3E, characteristic SWV peaks at ~−0.2 V were detected, with peak currents increasing as DSN assembly progressed, showing a rise that correlated with each additional layer of DSNs. This phenomenon is ascribed to the increased DNA concentration, which promotes the adsorption of MB, indicating the successful assembly of DSNs on the GO membrane. Overall, we have demonstrated a controllable method for fabricating tunable DSNs on the surface of the GO membrane by gradually increasing the number of assembled layers of multibranched DNA nanowires. Consequently, the membrane composition of DSNs could be accurately modulated for further investigating their effects on ion transport.

Before investigating the ion transport behavior, the surface charge of the DSN-*n*@GO membrane, which frequently exerts a notable influence on ion transport, was quantified. Because of the increasing negative charge from DSN-1 to DSN-5 structures, the surface charge of the bionic membrane could be precisely modulated by the assembly of DSN-*n*. The detailed relationship between the increasing layer of DSNs and the variation of surface charge is depicted in Fig. 3F. The ion transport behavior of the DSN-*n*@GO membrane was then studied using a two-electrode system with symmetric Ag/AgCl electrodes immersed in a 10 μM KCl solution at pH 7 (figs. S18 and S30). At this concentration of KCl, the charge shielding effect can be avoided because the double layer width formed at low concentrations, also known as the Debye length, can cover the entire DSN structure. The height of DSN-5 perpendicular to the GO surface is 90 nm (equivalent to 270 bases, with each three-base segment measuring 1 nm), which is less than the Debye length of 96.3 nm in a 10 μM KCl electrolyte solution (*62*–*65*). The results depicted in Fig. 2G illustrated the typical current-voltage (*I*-*V*) plots of the ssDNA@GO membrane before and after the programmable assembly of DSNs. Notably, when the ion current was focused at a bias of +1.0 V, a rise in current ranging from 0.20 to 0.65 μA was observed with each incremental layer of DSNs (Fig. 3G and fig. S31), indicating the precise regulation of ion current through the programmable modulation of the membrane composition. Moreover, the ion currents of DSN-*n*@GO membranes with varying layers of DSNs exhibited remarkable stability throughout the experimentation period (fig. S32). Furthermore, the ion current increase ratio [denoted as the (*I* − *I*_0_)/*I*_0_ value, where *I*_0_ and *I* are the currents of ssDNA@GO and DSN-*n*@GO at a bias of +1.0 V, respectively] gradually increased from 188 to 1 114% as the assembled DSNs from one layer to five layers (Fig. 3H), mirroring a similar trend to the alteration in the surface charge of DSN@GO. However, the ion current increase ratio was ~39% (fig. S33) when only R_1-1_ sequences containing β_0_ were added to the ssDNA@GO membrane due to the lower charge density of ssDNA. These results suggested that the surface charge of the membrane played a dominant effect on ion transport. To validate the effect of surface charge on the ion current increase ratio, numerical simulations based on the Navier-Stokes equations and the Poisson-Nernst-Planck equations were conducted (*66*). We used a 2D planar model to calculate ion transport in our biomimetic DSN-*n*@GO membrane system (fig. S34). As shown in Fig. 3I, following the assembly of DSN-*n* on the surface of the ssDNA@GO membrane, the simulated current increase ratio exhibited a similar increasing trend to our experimental findings. Thus, both the experimental and simulated results corroborated the predominant role of the surface charge of the DSN-*n*@GO membrane in regulating ion current. These results indicated the facile modulation of ion transport within the DSN-*n*@GO nanochannel system through programmable adjustment of membrane constituents, akin to a crucial characteristic observed in transmembrane pore proteins within cells.

To further explore the programmability and versatility of membrane composition modulation in our biomimetic nanochannel system, we integrated streptavidin-tagged phycoerythrin (PE-SA) and streptavidin-coated AuNPs (AuNP-SA) separately into the DSN to investigate the regulatory effects of proteins and inorganic metal particles as the membrane composition on ion transport. Specifically, the main backbone of nanowire-3 was tagged with biotin, facilitating tight binding to streptavidin. Thus, following the formation of DSN-3@GO, PE-SA or AuNP-SA could be tethered to the DSN-3@GO membrane (Fig. 3J). Because of their inherent negative charges, the introduction of PE-SA or AuNP-SA induced a greater current increase ratio compared to that observed with only DSN-3@GO (Fig. 3K). The current increase ratio caused by AuNP-SA exceeded that caused by PE-SA when using equivalent concentrations, attributed to the higher negative charge and the smaller size of AuNP-SA (Fig. 3, L and M). Hence, leveraging the programmability and addressability of DNA nanostructures, it becomes feasible to tailor the membrane composition with diverse nanomaterials and biomolecules, thereby regulating ion transport.

Motivated by the inactivation of the KcsA channel by POPG, we have developed a biomimetic DSN@GO membrane capable of precisely regulating transmembrane currents through the manipulation of the membrane composition. Various nanomaterials and biomolecules can be used as the composition of this bionic membrane to modulate ion transport due to the remarkable programmability and addressability of DSNs. Serving as an innovative biomimetic ion channel system, the DSN-*n*@GO membrane holds promise for catalyzing the advancement of the biomimetic systems.

### DSN content regulates membrane compositions

**Fig. 4.**
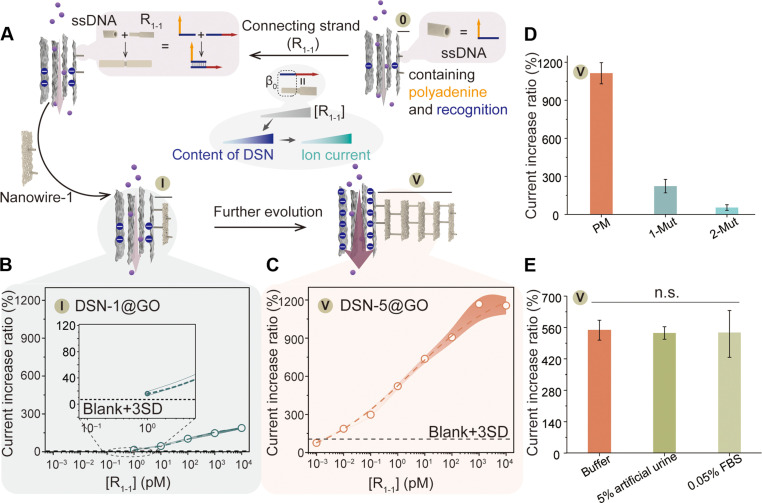
Regulation of DSN content on the ssDNA@GO membrane via the control of connecting strand. (**A**) Illustration of the modulation of DSN-1 or DSN-5 content on the ssDNA@GO membrane via varying amounts of the connecting strand. The current increase ratio measured in the (**B**) DSN-1@GO and (**C**) DSN-5@GO membrane upon addition of different concentrations of the connecting strand. The black dashed lines represent the minimal concentration of R_1-1_ for the modulation of DSN content based on the mean values of the blank sample plus three times the SD. Dashed lines with shaded areas, means ± SD (*N* = 3). (**D**) Current increase ratio of the DSN-5@GO membrane upon addition of 10 nM PM, 1-Mut, and 2-Mut connecting strand. The data represent the means ± SD (*N* = 3). (**E**) Ion current increase ratio of the DSN-5@GO membrane with 1 pM connecting strand in different matrices. n.s., not significant (*N* = 3).

After confirming the construction and the ability to modulate ion current of the biomimetic membrane with tunable membrane compositions by regulating the layers of DSNs, we proceeded to evaluate the impact of DSN content on regulating ion current. Given that the ion current increased with the surface charge of the GO membrane, we manipulated the DSN content by controlling the concentration of the connecting strand (R_1-1_) that connected the ssDNA@GO membrane with DSN nanostructures. Thus, the ion current could be arbitrarily tailored by adjusting the concentration of R_1-1_. As a proof-of-concept experiment, we selected DSN-1 and DSN-5 as models to investigate the regulatory effect of the connecting strand on ion transport performance (Fig. 4A and fig. S35). As illustrated in Fig. 4 (B and C), the ion current increase ratio [denoted as the (*I* − *I*_0_)/*I*_0_ value, where *I*_0_ and *I* are the currents of the nanochannels without and with connecting DNA R_1-1_ at a bias of +1.0 V, respectively] gradually increased with the concentration of R_1-1_ for the DSN-1@GO membrane, while the current increase ratio reached a plateau with more than 1 nM for the DSN-5@GO membrane, which can be attributed to the abundant negative charge and the limited concentration of DSNs. At equivalent R_1-1_ concentrations, the DSN-5@GO membrane exhibited a greater current increase ratio compared to the DSN-1@GO membrane (fig. S36). The minimal concentration of R_1-1_ capable of causing a discernible current increase ratio for both the DSN-1@GO and DSN-5@GO membrane was found to be 1 pM and 10 fM, respectively. The augmentation of the DSN content enhanced the surface charge, which is pivotal for ion transport, thereby leading to a larger ion current. Thus, the ion current could be programmatically regulated by manipulation of the concentration of the connecting strand and the architecture of DSNs.

Notably, owing to the high specificity of Watson-Crick base pairing, only the perfectly matched (PM) connecting strand demonstrated the ability to regulate the DSN-5 content on the ssDNA@GO membrane and induce an increase in ion current (Fig. 4D). Conversely, the single-base mutant (1-Mut) connecting strand and the two-base mutant (2-Mut) connecting strand failed to induce notable changes in the current increase ratio. Moreover, the connecting strand was also effective in adjusting the content of DSN-5 on the ssDNA@GO membrane in complex matrices, such as 5% artificial urine and 0.05% fetal bovine serum (FBS), without compromising the ratio of increase in ion current (Fig. 4E).

In summary, the DSN content can be precisely adjusted by varying the concentration of R_1-1_, allowing for the precise modulation of the ion current. Thus, we suggest that incorporating other nucleic acid biomarkers, such as microRNA (*67*), cell-free DNA (*68*), and mRNA (*69*), as connecting strands could demonstrate its potential for clinical applications.

### Reversible regulation of the membrane composition

**Fig. 5.**
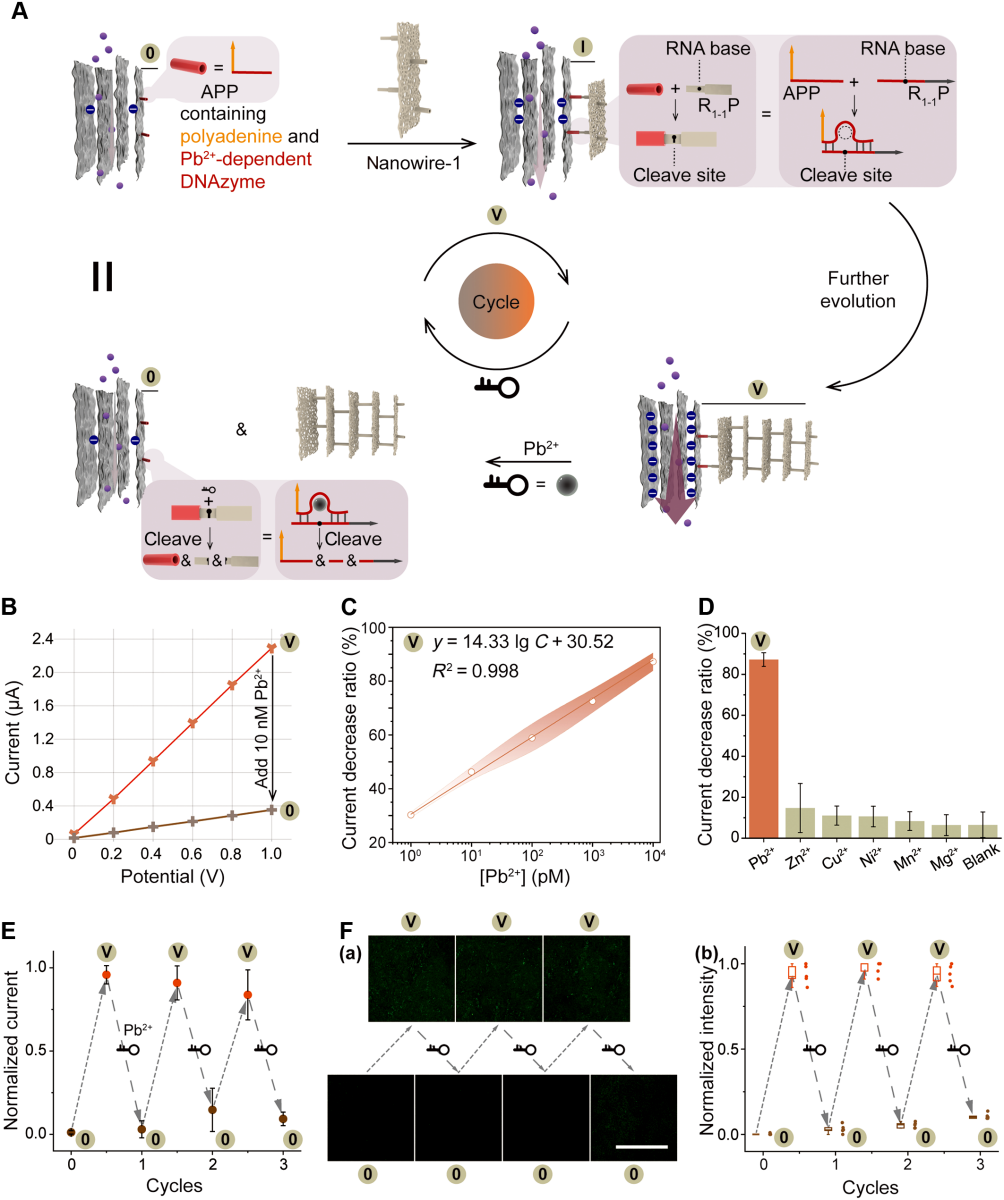
Reversible regulation of the DSN-5@GO membrane composition via integration of Pb^2+^-dependent DNAzyme. (**A**) Illustration of the release of DSN-5 nanostructures from the GO surface in the presence of Pb^2+^ and the subsequent generation of the DSN-5@GO membrane. (**B**) Typical *I*-*V* curves of the DSN-5@GO membrane without and with Pb^2+^. (**C**) Influence of varying Pb^2+^ concentrations on DSN-*n*@GO at a DSN-5 concentration of 10 nM. Higher concentrations of Pb^2+^ correspond to a greater current decrease ratio. Solid line with shaded area, means ± SD (*N* = 3). (**D**) The regulatory effect of Pb^2+^ and other divalent cations on DSN-5@GO demonstrated pronounced Pb^2+^ specificity, with the impact of other divalent cations on DSN-5@GO being nearly negligible. The data represent the means ± SD (*N* = 3). (**E**) Retrieval of ion current regulation by repeated assembly (orange symbols) and disassembly (brown symbols) processes. Downward arrow: 1 μM Pb^2+^ treatment. Upward arrow: 10 nM DSN-5 treatment. The data represent the means ± SD (*N* = 3). (**F**) Fluorescence confirmation of the reversibility of the DSN-5@GO membrane. (a) Confocal images of the reversible DSN-*n*@GO membrane (FAM-tagged multibranched DNA nanowires). Scale bar, 500 μm. (b) Statistical analysis of fluorescence intensity based on the corresponding data in (a). The data represent the means ± SD (*N* = 5).

The versatility of synthetic nucleic acids, particularly their binding and catalytic properties, prompted us to investigate the potential for releasing DSN-5 nanostructures from ssDNA@GO membranes to achieve the reversible regulation of the membrane composition. In this study, we devised a strategy wherein we redesigned the ssDNA (termed as APP) to include a polyadenine sequence for anchoring onto the GO surface and a Pb^2+^-dependent DNAzyme sequence for hybridization with a DNA-RNA chimeric substrate sequence (R_1-1_P) in nanowire-1 (fig. S37). Following the assembly of the DSN-5@GO membrane, the addition of Pb^2+^ ions induced the DNAzyme to fold into a secondary structure, leading to the cleavage of its substrate (*70*) and subsequent release of the DSN-5 nanostructures from the ssDNA@GO membrane (Fig. 5A). The ssDNA@GO membrane was capable of reassembling DSN nanostructures, thus regenerating the DSN-5@GO membrane. This design facilitated the reversible regulation of the membrane composition, allowing it to revert back to the ssDNA@GO state and regenerate the DSN-5@GO state. Consequently, this led to a decrease in ion current and an increase in ion current, respectively.

We initiated our investigation by examining the capability of Pb^2+^ for the reverse regulation of the DSN-5@GO membrane composition. As depicted in Fig. 5B, a notable reduction in ion current was observed when the DSN-5@GO membrane was incubated with 10 nM Pb^2+^, indicating the detachment of DSN nanostructures from the GO surface. Intriguingly, we observed a linear relationship between the concentration of Pb^2+^ and the ion current decrease ratio [denoted as the (*I*_0_ − *I*)/*I*_0_ value, where *I*_0_ and *I* are the currents of the nanochannels without and with Pb^2+^ at a bias of +1.0 V, respectively] (Fig. 5C), indicating that the detachment of DSN nanostructures was regulated by Pb^2+^. Because of the specificity of DNAzyme, only Pb^2+^ exhibited the capability to modulate the membrane composition and induce a decrease in ion current, whereas other divalent ions failed to elicit notable alterations in the current decrease ratio (Fig. 5D). Hence, by integrating Pb^2+^-responsive DNAzyme into DSN structures, we successfully achieved the reverse regulation of the DSN-*n*@GO membrane composition by varying the amounts of Pb^2+^, resulting in a decrease in ion current. We proceeded to investigate the regeneration of the DSN-5@GO membrane. Initially, the DSN-5@GO membrane was incubated with 1 μM Pb^2+^ to release DSN-5 nanostructures (state 0), followed by incubation with 10 nM DSN-5 nanostructures to regenerate the DSN-5@GO membrane (state V). Because of the surface charge variation between state 0 and state V, the normalized ion current transitioned from 0 to 1 and remained reversible for more than three cycles (Fig. 5E), which illustrated the capability of the DSN-*n*@GO membrane to achieve accurate and reversible control of ion current. To further elucidate the reason for this reversible modulation, we used FAM tagging of the multibranched DNA nanowires and conducted confocal laser scanning microscopy imaging of the membrane. As shown in Fig. 5F, a notable decrease in fluorescence intensity was observed upon the addition of 1 μM Pb^2+^ into the DSN-5@GO membrane, confirming the detachment of DSN nanostructures from the GO surface (state 0). Subsequently, the fluorescence intensity increased upon incubation with DSN-5 nanostructures, confirming that the ssDNA@GO membrane could hybridize with DSN-5 to regenerate the DSN-5@GO membrane (state V). The variation in fluorescence intensity of the bionic membrane between these two states was reversible for more than three cycles, illustrating the capability of the DSN-*n*@GO membrane to reversibly control the ion current derived from the regulation of the membrane composition. Besides, we propose that integrating other functional nucleic acids, such as aptamers, into DSNs could expand the range of molecules capable of regulating the biomimetic GO membrane composition. This approach holds promise for the development of versatile platforms for the precise manipulation of membrane properties in various applications.

## DISCUSSION

In conclusion, we have developed a biomimetic membrane featuring programmable and precise control over its composition in an artificial nanochannel system. Leveraging the programmability and addressability of DNA nanotechnology, we assembled tunable DSN nanostructures on the surface of the GO membrane to construct the DSN-*n*@GO membrane. Both experimental and simulated results have indicated the pivotal role of the surface charge of the DSN-*n*@GO membrane in governing ion transport. Our investigations have demonstrated the precise regulation of the membrane surface charge through adjusting the layers of DSNs, thereby finely modulating the ion current. Furthermore, we successfully integrated proteins and inorganic nanoparticles into DSN nanostructures to tailor membrane compositions to regulate ion transport. Notably, through the redesign of DSN-*n*@GO membranes containing DNAzymes, we achieved the reversible regulation of the membrane composition from the DSN-*n*@GO state to ssDNA@GO with the aid of Pb^2+^, resulting in a reduction in ion current. The ssDNA@GO membrane was capable of reassembling DSN nanostructures, thus regenerating the DSN-5@GO membrane and causing an increase in ion current.

While our demonstration of composition modulation in the bionic DSN-*n*@GO membrane for the precise regulation of ion transport is successful, there are opportunities for improvement in future investigations. For example, the current system lacks an in situ response to the connecting strand in undiluted complex matrices, which is important for its practical applications, such as biosensing. To address the high steric hindrance of proteins and other large biological molecules that affect DSN hybridization as well as viscosity influencing the test cell, we introduced a filtration step to dilute serum and urine. In future applications, efforts should focus on minimizing sample processing requirements before detection and preventing the adsorption of nontarget large biological molecules. In addition, dynamic monitoring of live cell metabolism would enhance the practical relevance of the current system. Prior studies demonstrated that flexible DSNs can manipulate cell-cell interactions (*54*, *71*). Thus, we envision introducing DSNs to facilitate interactions between a nanofluid and live cells, despite notable technical challenges. Further exploration should consider regulating the effective diameter of 1D nanochannels to manipulate multiple gating states, facilitating highly efficient on-demand cargo transport. Integrating responsive elements like i-motif, G-quadruplex, and aptamers onto DSNs holds promise for optimizing cargo transport efficiency in future studies (*72*–*74*). The successful construction of our bioinspired multiregulation nanochannel system would lay the foundation for the development of next-generation membranes, poised for diverse applications in diagnostics, separation, and energy conversion.

## MATERIALS AND METHODS

### Materials

Pristine GO nanosheets were purchased from XFNANO Co. Ltd. (Jiangsu, China). Artificial urine was purchased from Changfeng Technology Co. Ltd. (Guangdong, China). GelRed was purchased from Life-iLab Co. Ltd. (Shanghai, China). Streptavidin-labeled AuNPs was purchased from Biotyscience Co. Ltd. (Beijing, China). FBS was purchased from Gibco. All oligonucleotides (as shown in table S1) were synthesized and purified by Hippo Biotechnology Co. Ltd. (Beijing, China). Other chemicals were obtained from Sinopharm Chemical Reagents Co. Ltd. (Shanghai, China) and used without further purification. All solutions were prepared with Milli-Q water with a resistivity of 18.2 megohm/cm. The data plots are processed and visualized using an online platform: ChiPlot (https://chiplot.online).

### Preparation of multibranched DNA nanowires

For the preparation of nanowire-1, DNA S_1_ and S_2_ were mixed in a reaction buffer [10 mM tris-HCl and 120 mM NaCl (pH 7.4)] and then, after heating at 95°C for 10 min, slowly cooled to room temperature. DNA R_1-1_ and R_2-1_ were individually snap cooled in a 1× reaction buffer by heating at 95°C for 10 min and incubating at 4°C for 7 min. Last, S_1_ and S_2_ and R_1-1_ and R_2-1_ are mixed together and hybridized for 1 hour. The branches of other nanowires (nanowire-2, nanowire-3, nanowire-4, and nanowire-5) are made from (R_1-2_ and R_2-2_), (R_1-3_ and R_2-3_), (R_1-4_ and R_2-4_), and (R_1-5_ and R_2-5_), respectively.

### Preparation of DSNs

In test tube experiments, the DSN-*n* network can be assembled step by step from a low-numbered DNA structure to a high-numbered DNA structure or reacted in a one-pot manner. All reaction experiments are conducted in a hybridized buffer [10 mM PB, 20 mM Mg^2+^, and 1 M NaCl (pH 7.4)]. In nanochannel experiments, ssDNA is first loaded on the membrane surface. Subsequently, DNA modules are introduced step by step to fabricate the DSN-*n* network.

### Calculation of the membrane surface charge density

The GO membrane charge density (σ, mC m^−2^) is determined by the membrane zeta potential using the Gouy-Chapman equation, which is expressed as follows (*33*, *75*)

$$\sigma =-\varepsilon \kappa \xi \frac{\text{sinh}\left(\frac{F\xi }{2\mathrm{RT}}\right)}{\frac{F\xi }{2\mathrm{RT}}}$$ (1)

where $\kappa^{-1}={\left(\frac{\varepsilon \mathrm{RT}}{2F^{2}C}\right)}^{\frac{1}{2}}$ is the Debye length, ξ (mV) is the membrane zeta potential obtained through streaming potential measurements, *R* = 8.3145 J mol^−1^ K^−1^ is the gas constant, *F* = 96,485 C mol^−1^ is the Faraday constant, *T* = 298 K is the absolute temperature, and ε = 6.933 × 10^−10^ F m^−1^ is the permittivity. When the potential is low, Eq. 1 can be simplified as follows

$$\sigma =-\frac{\varepsilon \xi }{\kappa^{-1}}$$ (2)

The DSN-*n*@GO membrane charge density is the sum of the charge density of the GO membrane and that of DSN-*n*. The surface charge variation induced by DSN-*n* is determined through ruthenium ion adsorption tests conducted over an area of 0.49 cm^2^.
