# Supplementary material for: Precise control of transmembrane current via regulating bionic lipid membrane composition
Source: Sci Adv. 2024 Aug 30;10(35):eadq0118. doi: 10.1126/sciadv.adq0118 (PMC11364097; doi:10.1126/sciadv.adq0118)
Supplement: Supplementary file 1 — Supplementary Materials and Methods Figs. S1 to S37 Tables S1 to S3 [file sciadv.adq0118_sm.pdf]

Science Advances
AAAS

Supplementary Materials for

Precise control of transmembrane current via regulating bionic lipid membrane composition

Zhiwei Shang *et al.*

Corresponding author: Meihua Lin, linmh@cug.edu.cn; Fan Xia, xiafan@cug.edu.cn

Sci. Adv. **10**, eadq0118 (2024)
DOI: 10.1126/sciadv.adq0118

This PDF file includes:

Supplementary Materials and Methods
Figs. S1 to S37
Tables S1 to S3

Supplementary Materials and Methods

Membrane preparation. 40 mg pristine graphene oxide (pGO) powder was dissolved in 40 mL ultrapure water followed by ultrasonication for 6 h to obtain the pGO dispersion. Then 80 mg tris (hydroxymethyl) aminomethane was dissolved in 40 mL pGO dispersion followed by adjusting the pH to 8.5 through adding 2 M HCl dropwise. Then, 40 mg DA was added in the aforementioned pGO dispersion under stirring at room temperature for 60 min to obtain polydopamine (PDA)-modified graphene oxide (GO) dispersion. Using vacuum filtration, drying and curing in an oven at 60°C.

Agarose gel electrophoresis. 1% agarose gels, stained with Gelred (Life-iLab), were used for agarose gel electrophoresis.10 μL DNA sample and 2 μL 6×loading buffer was mixed and used as sample solution. Electrophoresis was performed in fresh 1×Tris-borate-EDTA buffer (1×TBE, 90 mM Tris, 90 mM boric acid, and 10 mM EDTA, pH 8.0) at 90 V for 90 min in an ice-water bath. After that, The Bio-Rad ChemiDoc XRS System was used to image and analyze all agarose gels.

Atomic force microscopy (AFM) imaging. 15 μL DNA sample solution and 5 μL $NiCl_2$ (300 μM) was mixed for 6 min to enhance the adhesion of DNA samples on the surface of mica. The mixture of 10 μL solution was deposited on freshly cleaved mica and left for 8 min. After that, DNA sample was rinsed with ultrapure water 3-5 times and dried in nitrogen atmosphere. Multimode 8 (Bioscopesystem, Bruker, USA) was used to image the DNA samples using the ScanAsyst mode. NanoScopeAnalysis was used to analyze the images.

Zeta potential in solution. The zeta potentials of DSN-n, pGO nanosheet, GO nanosheet, streptavidin-tagged phycoerythrin (PE-SA) and streptavidin-coated gold nanoparticles (AuNP-SA) were evaluated using Nano-ZS90 (Malvern, UK). The zeta potential measurements of GO and pGO nanosheets were performed at pH 7 (in water). The other samples were diluted in hybridization buffer (10 mM PB, 20 mM Mg^{2+}, 1 M NaCl, pH=7.4). Parallel data for AuNP-SA and PE-SA were collected 10 times, while other samples were collected 30 times in parallel. All samples were measured at 25 ℃.

Fabrication of 2D nanofluidic device. To fabricate the 2D nanofluidic device, the GO membrane was cut into a rectangular piece (length (l) ×width (w), 10 mm ×6 mm) that was then sandwiched between two pieces of polydimethylsiloxane (PDMS) elastomers using a blend of PDMS prepolymer and reserving either side of the rectangular GO membrane, followed by filling with 10 μM KCl for 8h.

Electrochemical impedance spectroscopy (EIS). EIS was carried out by a three-electrode system, with the gold-plated electrodes that modified the DSN-n as working electrode, an Ag/AgCl as reference electrode and a platinum wire as auxiliary electrode. The EIS measurement solution was 5 mM $Fe(CN)_6^{3-/4-}$ containing 0.1 M KCl (pH=7.0). The AC voltage amplitude was 5 mV, and the voltage frequencies ranged from 0.01 Hz to 100 kHz.

Electrolytic current measurements. The GO membrane was incubated with 1 μM ssDNA containing poly-adenine sequence and recognition sequence in hybridization buffer (10 mM PB, 20 mM Mg^{2+}, 1 M NaCl, pH=7.4) for 30 min. After washing with hybridization buffer, the ssDNA@GO membrane was incubated in 3% BSA solution for 1 h to block excess nonspecific binding sites on the surface. Subsequently, DNA modules are introduced step by step to fabricate the DSN-n@GO membrane. For studies of the MB-DNA binding, the DSN-n@GO membranes were immersed for 20 min in 1 mM solution of methylene blue (MB) followed by washing with hybridization buffer. Electrolytic current acquisition was carried out on a CHI 1040C electrochemical workstation (Shanghai, China). In SWV, a modulation amplitude was 50 mV, a step potential was 4 mV and a frequency was 10 Hz.

Confocal laser scanning microscope. The fluorescence signals were detected by using a Zeiss LSM 880 confocal microscope. Confocal images and the fluorescence intensity of nanochannel were obtained by 10 × plan apochromatic objective (NA=0.45, WD=2.1 mm). In the dual-channel mode, the FAM was excited by a laser at 488 nm, and was collected in the range of 489-530 nm. A 633 nm laser was used to excite CY5, which was collected at 640-735 nm.

Typical current−voltage (*I*−*V*) measurements. The $I-V$ curves through the nanochannels were obtained using a sourcemeter unit (Keithley 2401), with two bare Ag/AgCl reference electrodes inserted into each bulk electrolyte reservoir. Steady-state conductance measurement is necessary to obtain reliable results. $I-V$ curves under different DNA scaffold networks labeling ranging from DSN-1 to DSN-5 were recorded at an applied voltage of −1.0 to 1.0 V after the 2D nanofluids were further soaked in the 10 μM KCl for 8 h.

Numerical simulation. Continuum dynamics is utilized to examine ion transport within nanofluidic channels. The integrated Poisson-Nernst-Planck (PNP) equations and Navier-Stokes (NS) equations are applied for computing the ion current within these channels.
The transport properties of charged nanopores can be described for each ion species by Nernst-Planck equation as follow,

$$J_i = -D_i\left(\nabla c_i + \frac{z_i e c_i}{k_B T}\nabla\varphi\right) \qquad i = +,- \tag{3}$$

Here, i stands for the ion specie, J_i is the local ion flux, D_i is the diffusion coefficient, c_i is the local ion concentration, z_i is the valence, φ is the local electrical potential, k_B, T, e represent respectively for the Boltzmann constant, the temperature, the electron charge as their usual meanings. In the nanochannel, φ is determined by the Poisson equation:

$$\nabla^2\varphi = -\frac{1}{\varepsilon}\sum_i z_i e c_i \qquad i = +,- \tag{4}$$

Where ε is the dielectric constant of the electrolyte solution. In the continuous electric field, the steady-state solution could be calculated as:

$$\nabla\cdot(c_i u + J_i) = 0 \qquad i = +,- \tag{5}$$

The solution is considered incompressible. Therefore, the velocity vector of the solution gradient, $\nabla\cdot u$ is equal to zero. The complete set is the Navier-Stokes equation:

$$u\nabla\cdot u = \frac{[-\nabla p + v\nabla^2 u - (c_+ - c_-)\nabla\varphi]}{\rho} = 0 \tag{6}$$

Where p is pressure, v is the solution viscosity and ρ is the mass density of solution, and c_+ and c_- are the concentrations of K^+ and Cl^-, respectively. In this study, it is supposed that there is no

pressure gradient, which represents ∇p is equal to zero. The ion current, I, across the nanochannel can be calculated by the Poisson, Nernst-Planck and Navier-Stokes (PNP-NS) equations:

$$I = \iint J_i dS = -\iint D_i \left(\nabla c_i + z_i c_i \frac{F}{RF} \nabla \varphi\right) dS \quad (7)$$

Where F is the Faraday constant.

Supplementary Figures

Design and characterization of tunable DSN

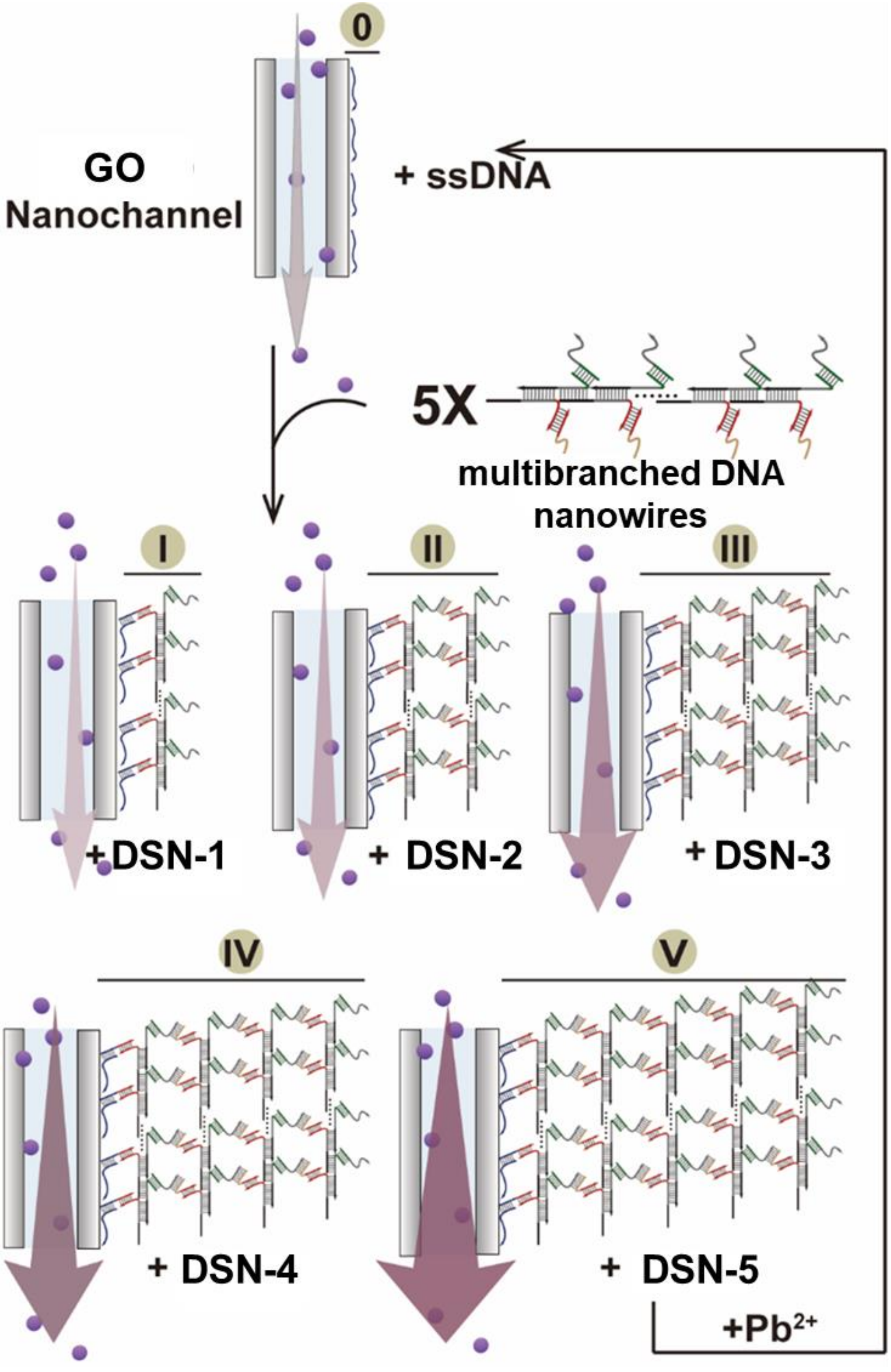

Fig. S1. Schematic representation of DSN modulating ion current within the nanochannel.

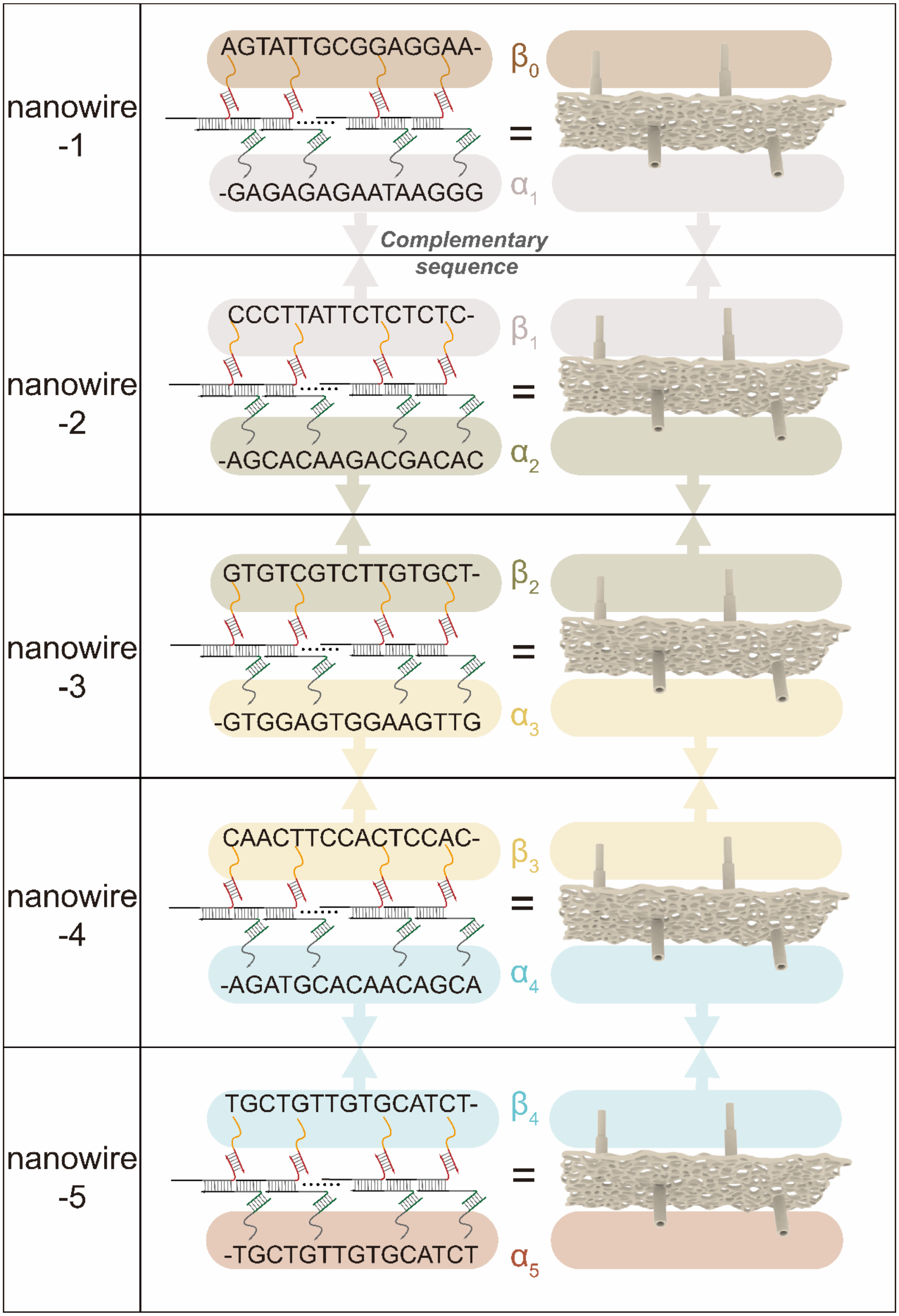

Fig. S2. Schematic representation of multibranched DNA nanowires and sequence of α_n and β_n.

The main structure of the multibranched DNA nanowires is formed by the partial complementary hybridization of two single-stranded DNA, S_1 and S_2, serving as the backbone. The non-complementary regions hybridize with the R_{1-n} and R_{2-n} partially, forming branches of the DNA nanowire. The exposed sticky ends on the branches are named as α_n and $\beta_{(n-1)}$. Through the complementary hybridization of α_n and β_n, the multibranched DNA nanowires are coupled, generating the DSN structure.

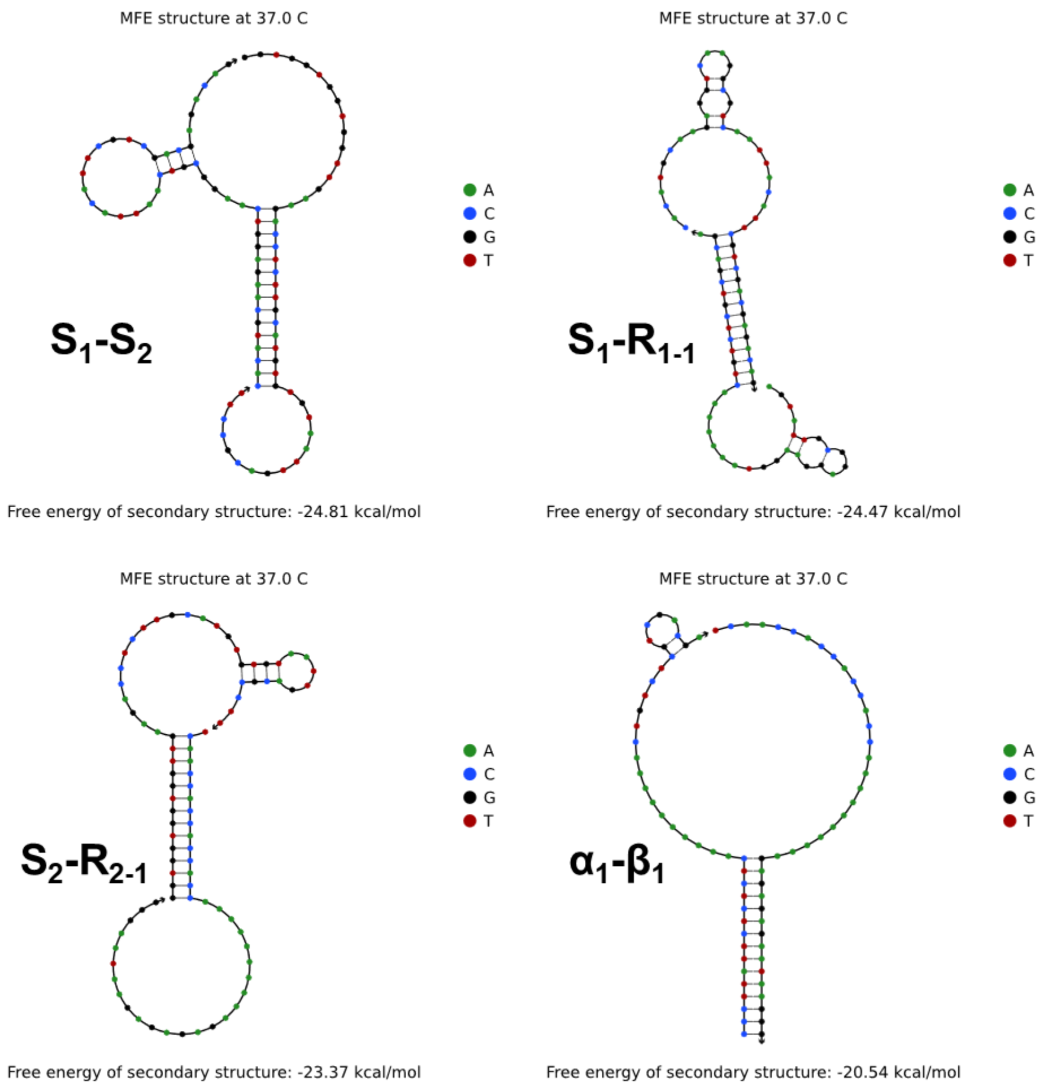

Fig. S3. ΔG values of hybridization chain reaction of DNA branched polymer were calculated, with oligonucleotides analyze tool from NUPUCK.
NUPACK: Nucleic Acid Package

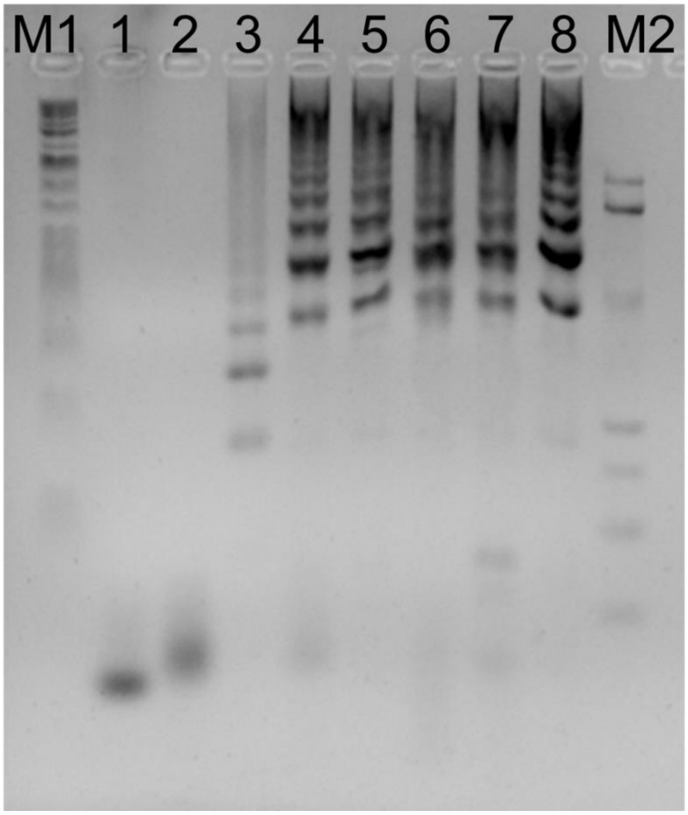

Fig. S4. AGE analysis of multibranched DNA nanowires. From lane 1 to lane 8: S_1; R_{1-1}; S_1+S_2; nanowire-1; nanowire-2; nanowire-3; nanowire-4; nanowire-5. M1: DNA marker (50-1500 bp). M2: DNA marker (25-500 bp).

In lane 3, the supersandwich structure formed by the hybridization of S_1 and S_2 is clearly visible. With the addition of branch strands (from lane 4 to lane 8), there is a notable increase in molecular weight, indicating successful hybridization of the branch strands onto the main chain to form the multibranched DNA nanowire structure (nanowire-1 to nanowire-5).

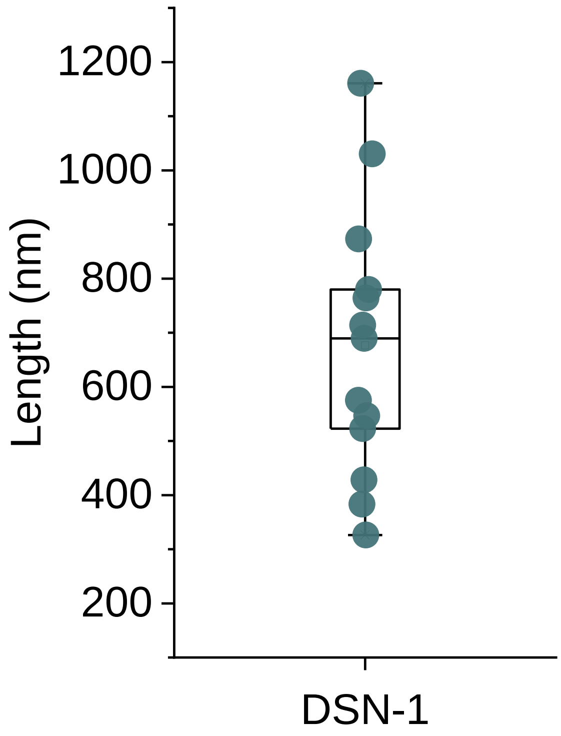

Fig. S5. Distributions of the length of DSN-1, measured from the SEM image (N=13).

Performance and stability of GO membrane

When a pristine graphene oxide (pGO) membrane is immersed in an aqueous solution, an abundance of oxygenated functional groups present on pGO nanosheets promotes strong hydration, thereby facilitating the capture of excessive water molecules within the interlayer channels of pGO. The presence of additional water molecules absorbed within the pGO nanochannels can cause undesired swelling, resulting in an increase in interlayer spacing and disturbance of the well-stacked pGO laminates intended for selective water transport. Based on the captivating characteristics of polydopamine (PDA), which combines strong binding reactivity of the fatty amine groups and appropriate molecular configuration with high steric hindrance, we selected PDA as a molecular bridge to regulate the stacking of graphene oxide (GO) and create stable nanochannels (fig. S6). The dispersive solution of GO exhibited a darker appearance compared to the similarly concentrated pGO (fig. S7 and S8). Fig. S9 displays AFM scanning images of pGO and GO nanosheets. Following PDA modification, multiple layers nanosheets are bonded together, resulting in a significant increase in the height of the nanosheets. Following functionalization with PDA, the zeta potential experiences an increase from around -35 mV to approximately -30 mV, attributable to the positive charge conferred by PDA (fig. S10).

The obtained PDA-modified pGO dispersion was subsequently filtrated, dried, and processed into a flexible and self-supporting GO membrane (fig. S11). The presence of amide (C-N) absorption peaks in the Fourier transform infrared (FTIR) spectra (fig. S12) provides evidence for the formation of chemical bonds between the amine monomer and pGO nanosheets. These bonds result from condensation reactions with carboxyl groups and nucleophilic addition reactions with epoxy groups. fig. S13 illustrates that the GO composite (II & IV) exhibits an elevated nitrogen content, indicative of reactions between amine monomers and pGO nanosheets. These reactions lead to polymerization and the formation of the GO composite, aligning with the FTIR results discussed earlier. As observed in the X-ray photoelectron spectroscopy (XPS) results of the stacked laminates, a new peak representing C=N bonding appeared at 285.7 eV, and O-C=O peak decreased obviously due to the reaction of DA with the carboxyl groups on pGO, indicating the reaction between pGO and DA (fig. S14). The stability test shows that GO is more stable in water than pGO membrane and can maintain stability for a week (fig. S15). Moreover, the 6 μm thick GO membrane preserves the gap between the stacked nanosheets, providing a channel for ion transport (fig. S16). Compared to pGO membranes, the GO membrane exhibits only slight 2θ shifts in water, corresponding to minimal interlamellar spacing (d-spacing) variation from ~0.94 to 0.97 nm (fig. S17). Therefore, it possesses the attractive anti-swelling capability in aqueous environments. Additionally, electrochemical tests were used to verify its stability (fig. S18 and S19). After 30 consecutive electrochemical current–voltage (*I-V*) tests, the signal values were not significantly different, indicating that the film was stable enough to be used in subsequent experiments.

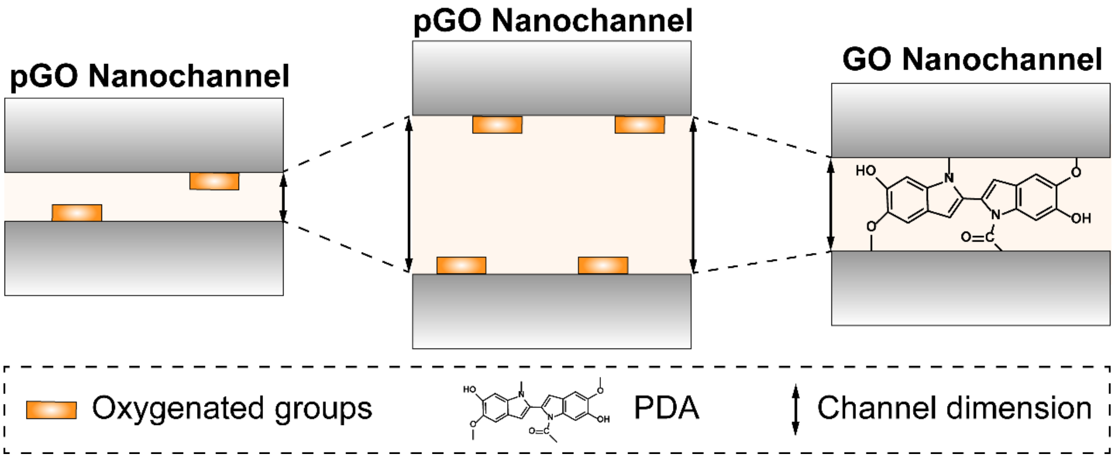

Fig. S6. Schematic diagram of various GO membrane nanochannel dimensions.
The reduction of pGO eliminates its oxygenated functional groups and reduces the dimensions of the interlayer nanochannel between adjacent nanosheets, thereby enhancing confinement. Conversely, the molecular configuration with high steric hindrance of PDA within the laminates exerts an opposing influence on the channel dimensions.

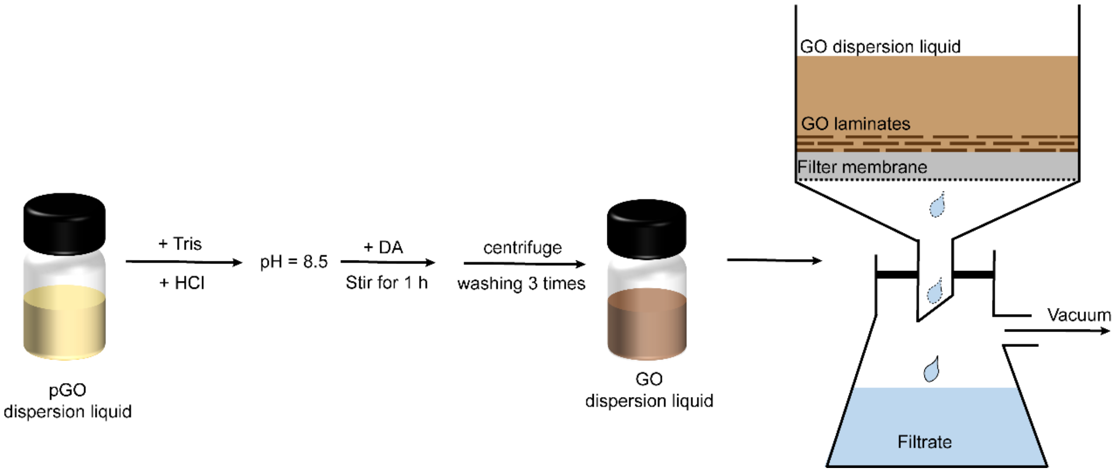

Fig. S7. The synthesis of GO membrane.
The membrane preparation progress is depicted in fig. S6. In brief, the dispersion of pGO was adjusted to a pH of 8.5, followed by the addition of dopamine hydrochloride (DA) and stirring at room temperature for 1 hour. Then the dispersion was washed by ultrapure water 3 times. The GO nanosheets were vertically stacked using vacuum filtration.

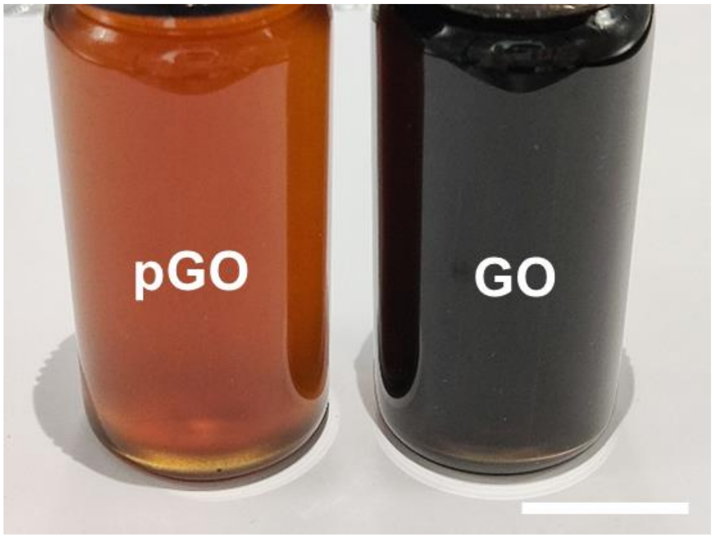

Fig. S8. Photos of dispersions for pGO and GO. Scale bar: 2 cm.
Due to the polymerization of PDA, the color changes from tan to black.

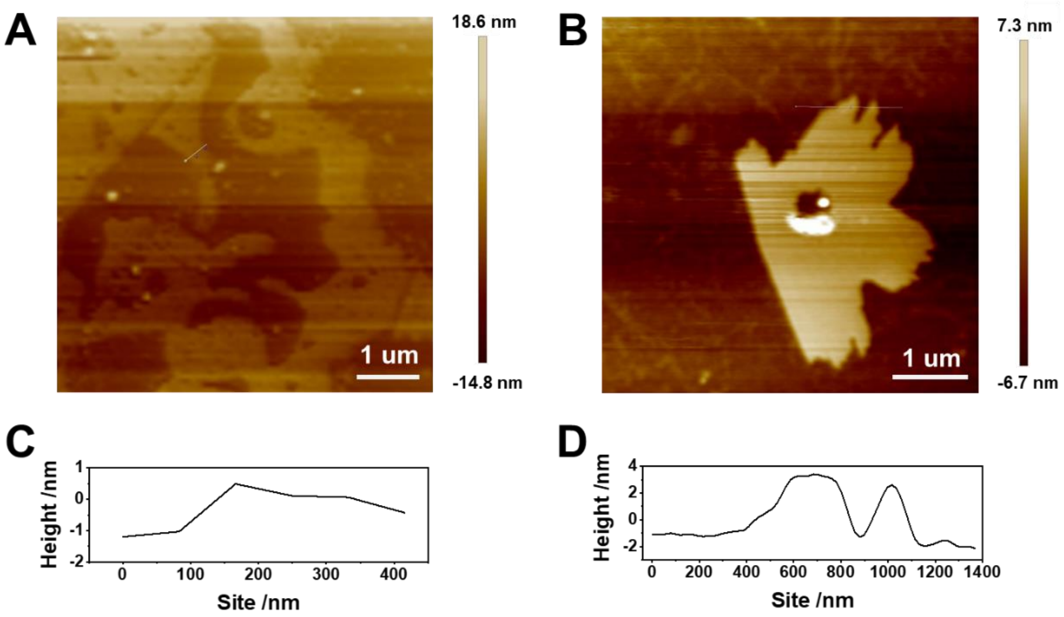

Fig. S9. AFM imagine and corresponding height statistical lines of pristine pGO (A, C) and GO (B, D) nanosheets. Scale bar: 1 µm.

Due to the crosslinking effect of PDA, multiple layers of nanosheets are crosslinked together, resulting in an increase in the height values measured by AFM.

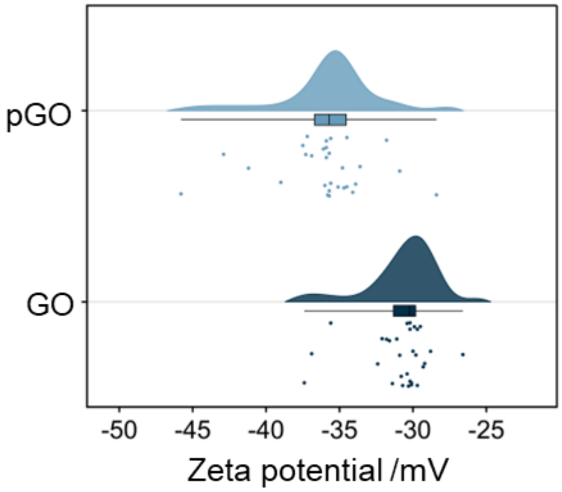

Fig. S10. Scatter dot plots showing the zeta potential for pGO and GO dispersion measured at pH 7. (N = 30 repeats).

After functionalization with PDA, the zeta potential increases from approximately -35 mV to approximately -30 mV due to the positive charge of PDA. The pH of the GO and pGO dispersion liquids was adjusted to 7 using tris and HCl before the test.

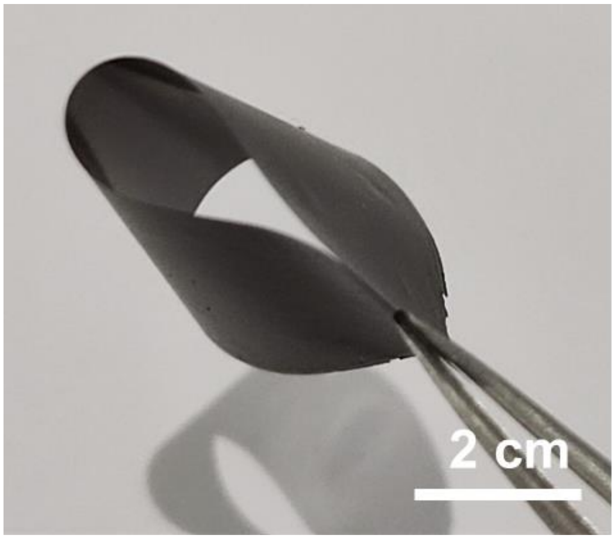

Fig. S11. Photographs of a flexible and self-supporting GO membrane. Scale bar: 2 cm.

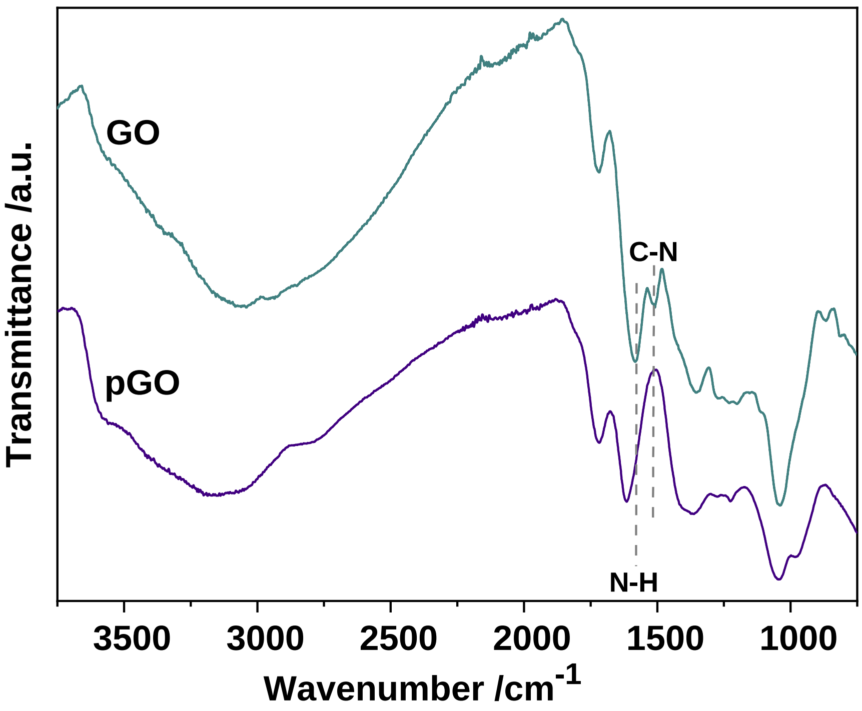

Fig. S12. FTIR spectra of pGO and GO membranes.

The pGO laminate displayed distinctive peaks corresponding to the stretching vibrations of hydroxyl (-OH), carboxyl (C=O), carboxy (-COOH), alkoxy (C-OH), and epoxy (C-O-C) groups at wavenumbers approximately around 3240 cm^{-1}, 1740 cm^{-1}, 1370 cm^{-1}, 1220 cm^{-1}, and 1030 cm^{-1}, respectively. These findings are consistent with previous investigations in the field. Upon the introduction of PDA, the intensity of the carboxy group declined as a result of its consumption in the reactions. In addition to the characteristic bands originating from pGO, novel absorption peaks were detected at approximately 1560 cm^{-1} and 1500 cm^{-1}, corresponding to the presence of amine (N-H) and amide (C-N) functionalities, respectively. These findings provide evidence that the amine monomers underwent chemical interactions with the oxygenated groups of pGO nanosheets, leading to the formation of chemical linkages through condensation and nucleophilic addition reactions.

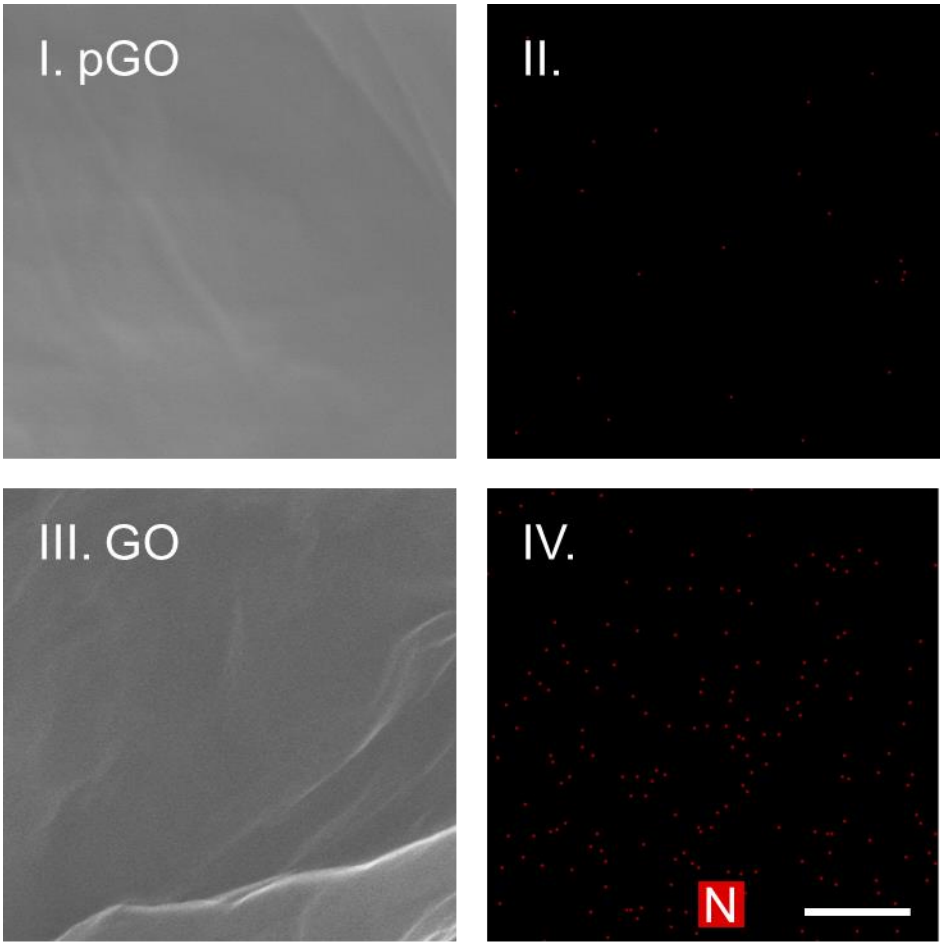

Fig. S13. Energy dispersive spectroscopy of the N (in red) elements from pGO and GO membrane. Scale bar: 500 nm.

The higher nitrogen content observed in the GO membrane, containing PDA (II &IV), suggests that the amine monomers reacted with the pGO nanosheets.

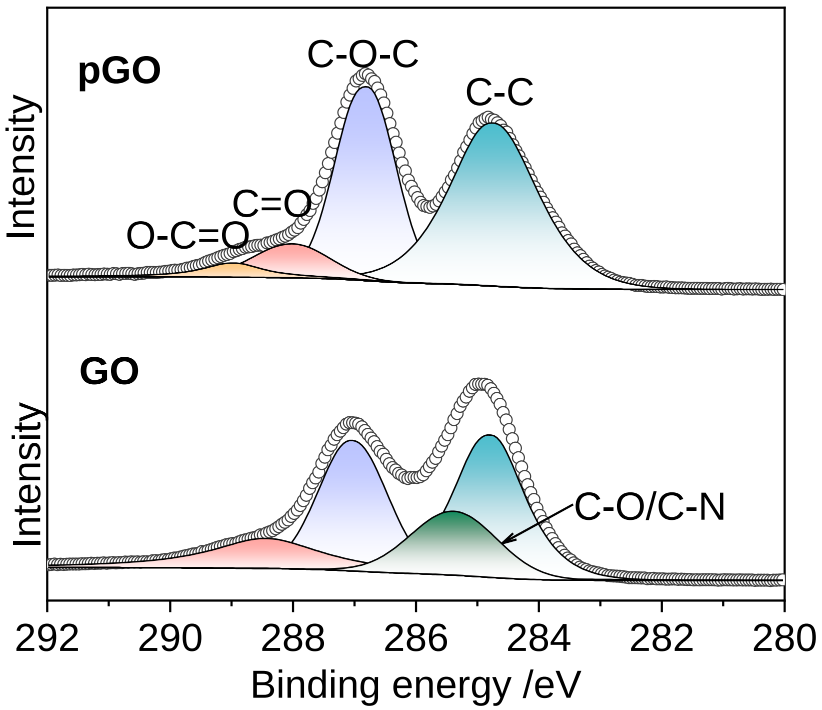

Fig. S14. XPS spectra of pGO and GO membrane.

The pGO curve exhibits four peaks corresponding to distinct chemical bonds: C=C (284.9 eV), C=O (286.9 eV), C=O (288.2 eV), and O=C-O (289.3 eV). In contrast, GO shows an additional peak attributed to C-N bonding at 285.7 eV, signifying the reaction between DA and pGO.

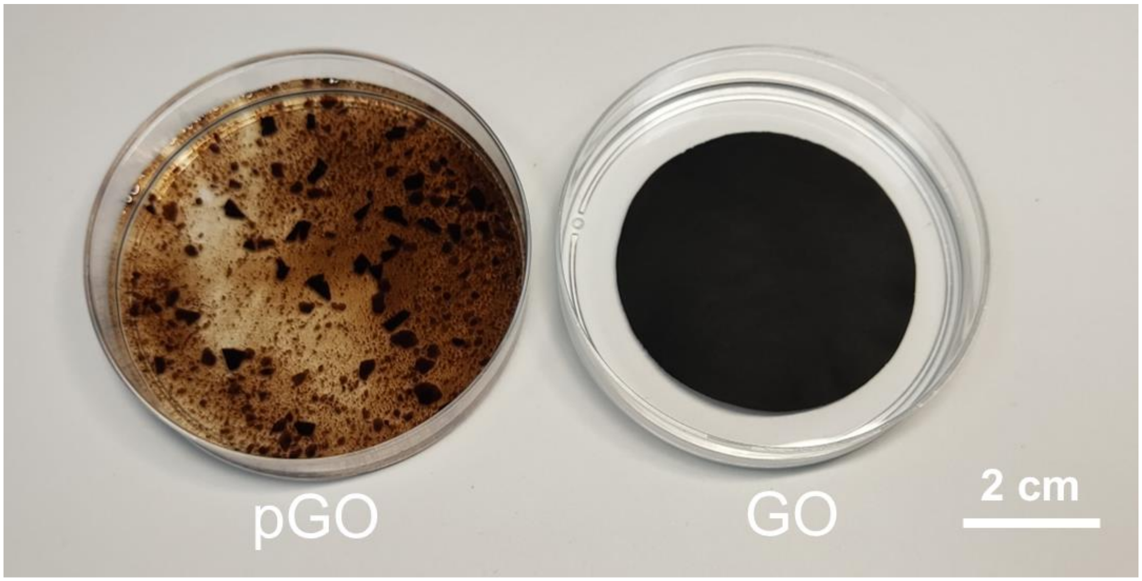

Fig. S15. Stability test results of pGO and GO membrane in water, after soaking in water for 1 week and stirring the water once. Scale bar: 2 cm.

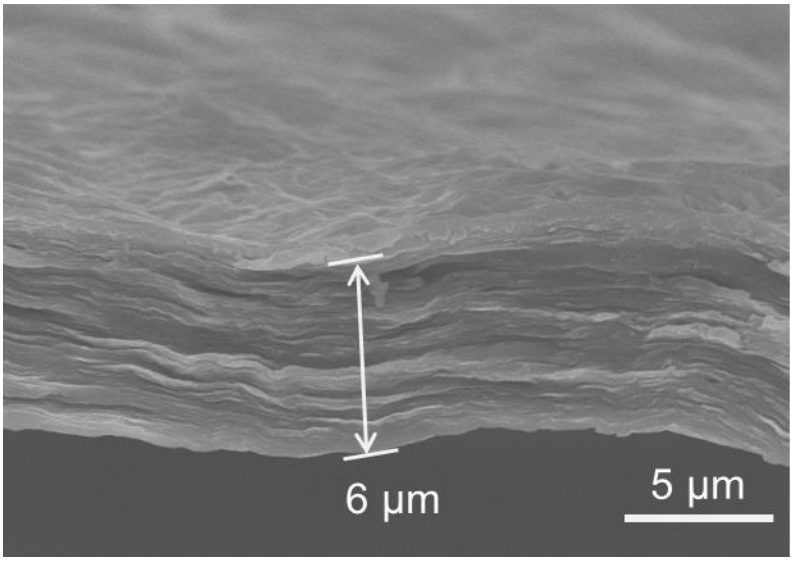

Fig. S16. Cross-section view scanning electron microscopy (SEM) images of GO membrane. Scale bars: 5 µm.

The layered GO membrane has a thickness of 6 µm, with gaps between the layers.

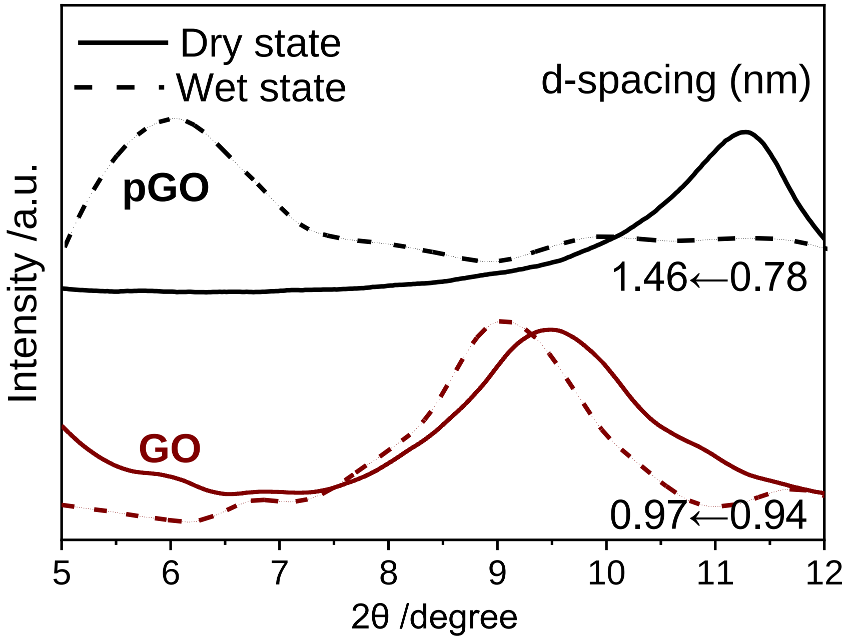

Fig. S17. XRD of pristine pGO (black line) and GO (red line). Solid line: dry state; dotted line: wet state.

After functionalizing with PDA, the interlamellar spacing (d-spacing) increases from ~0.78 nm to ~0.94 nm. Nonetheless, the GO membrane displayed only a marginal 2θ shift in water, indicative of a minimal expansion in d-spacing from ~0.94 to ~0.97 nm. This suggests its superior resistance to swelling in aqueous environments, making it well-suited for experimental applications in such conditions.

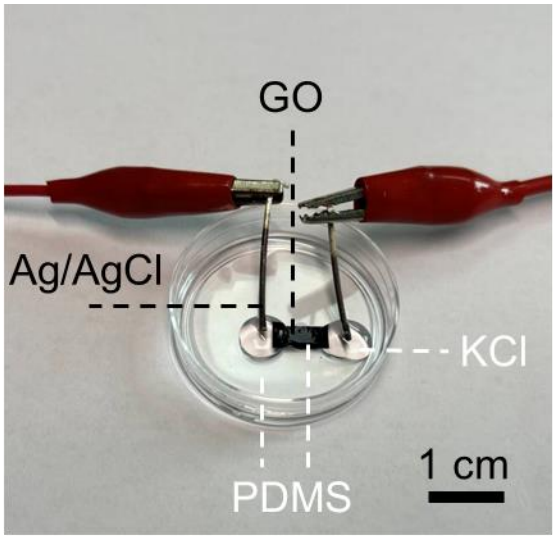

Fig. S18. Optical image of the nanochannel device.
The symmetric GO membrane was placed between two pieces of polydimethylsiloxane (PDMS) elastomers by using a mixture of PDMS prepolymer, with both sides of the rectangular GO membrane being protected. The ends of the GO membrane were left exposed to the 10 μM KCl electrolyte. Scale bar: 1 cm.

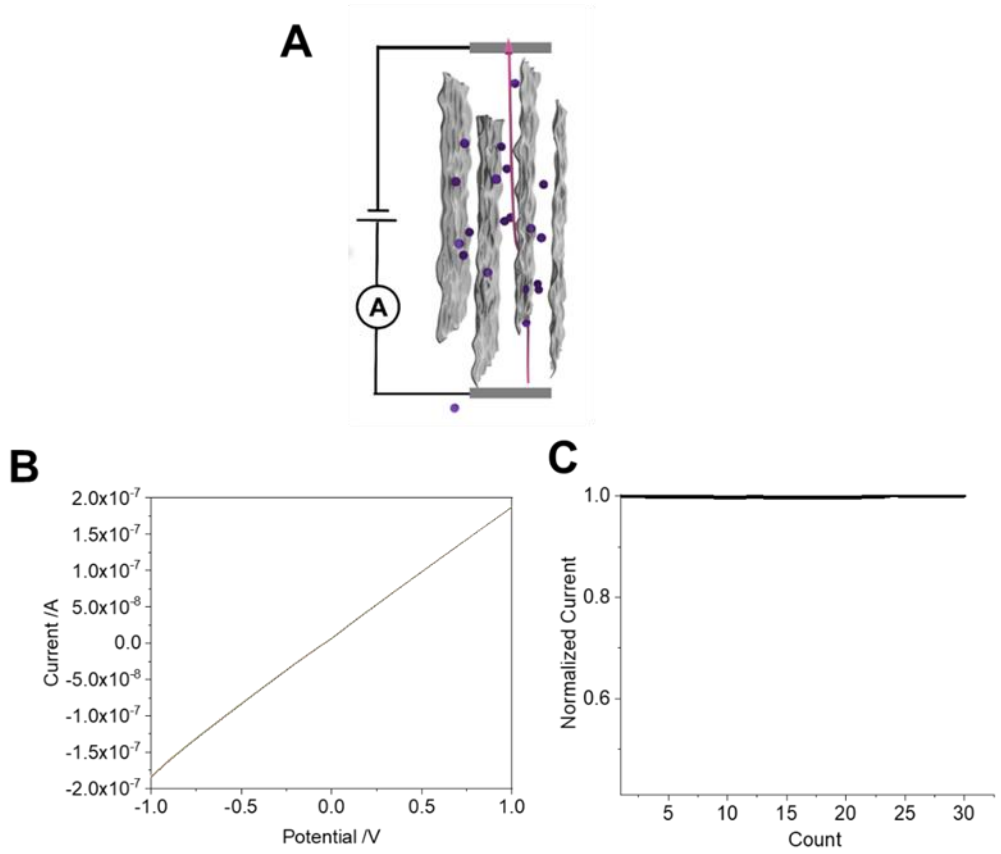

Fig. S19. The stability of GO membrane was investigated by *I-V* tests with 30 continuous repeats. (**A**) Setup for ion signal measurement of GO membrane. (**B**) *I*−*V* curves from the 1st scan to 30th scan were obtained from GO membrane. (**C**) The stability of the GO membrane was assessed by examining the normalized ion current. The normalized ion signals at +1.0 V were derived from the corresponding *I-V* curves.

Construction and characterization ssDNA@GO membrane

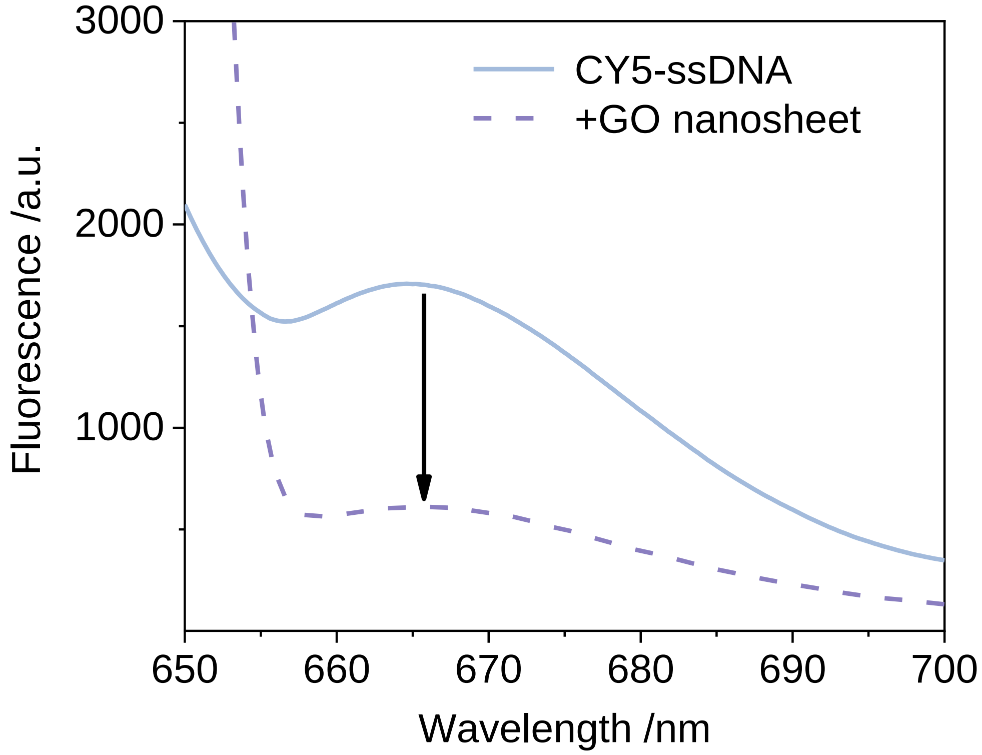

Fig. S20. The fluorescence measurement of CY5-ssDNA before (solid line) and after (dotted line) adding GO nanosheets.

Upon the adsorption of CY5-ssDNA onto the GO surface, a notable reduction in fluorescence intensity occurs due to the strong fluorescence quenching properties of GO.

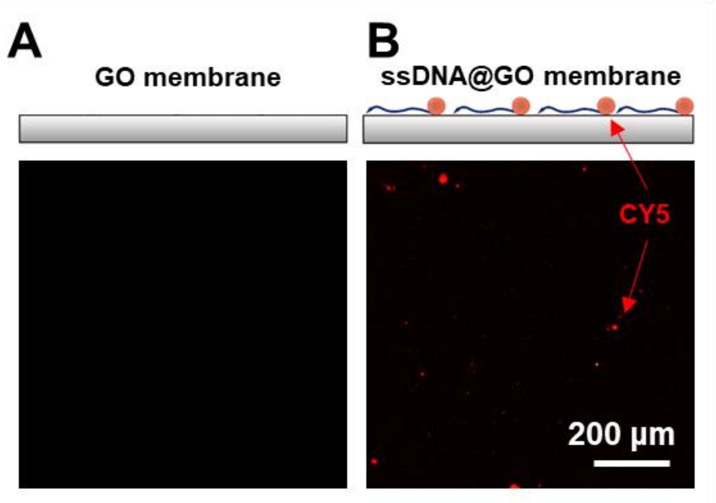

Fig. S21. Confocal microscopy was employed to visualize the (A) GO membrane and (B) ssDNA@GO membrane. The ssDNA was tagged with CY5. Scale bar: 200 μm.

The images clearly depict a slight fluorescence signal on ssDNA@GO membrane owing to the presence of the CY5-tagged ssDNA, while no fluorescence signal is observed on GO membrane. This confirms the successful immobilization of the ssDNA on the GO membrane, indicating the effectiveness of the synthesis process of ssDNA@GO.

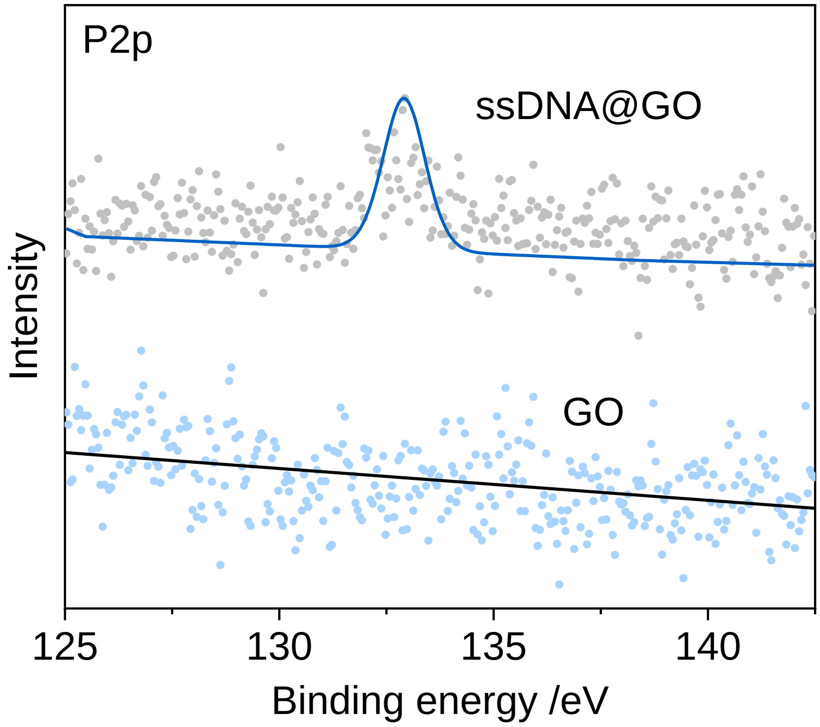

Fig. S22. XPS spectra of the P2p region for GO (down) and (up) ssDNA@GO membrane.

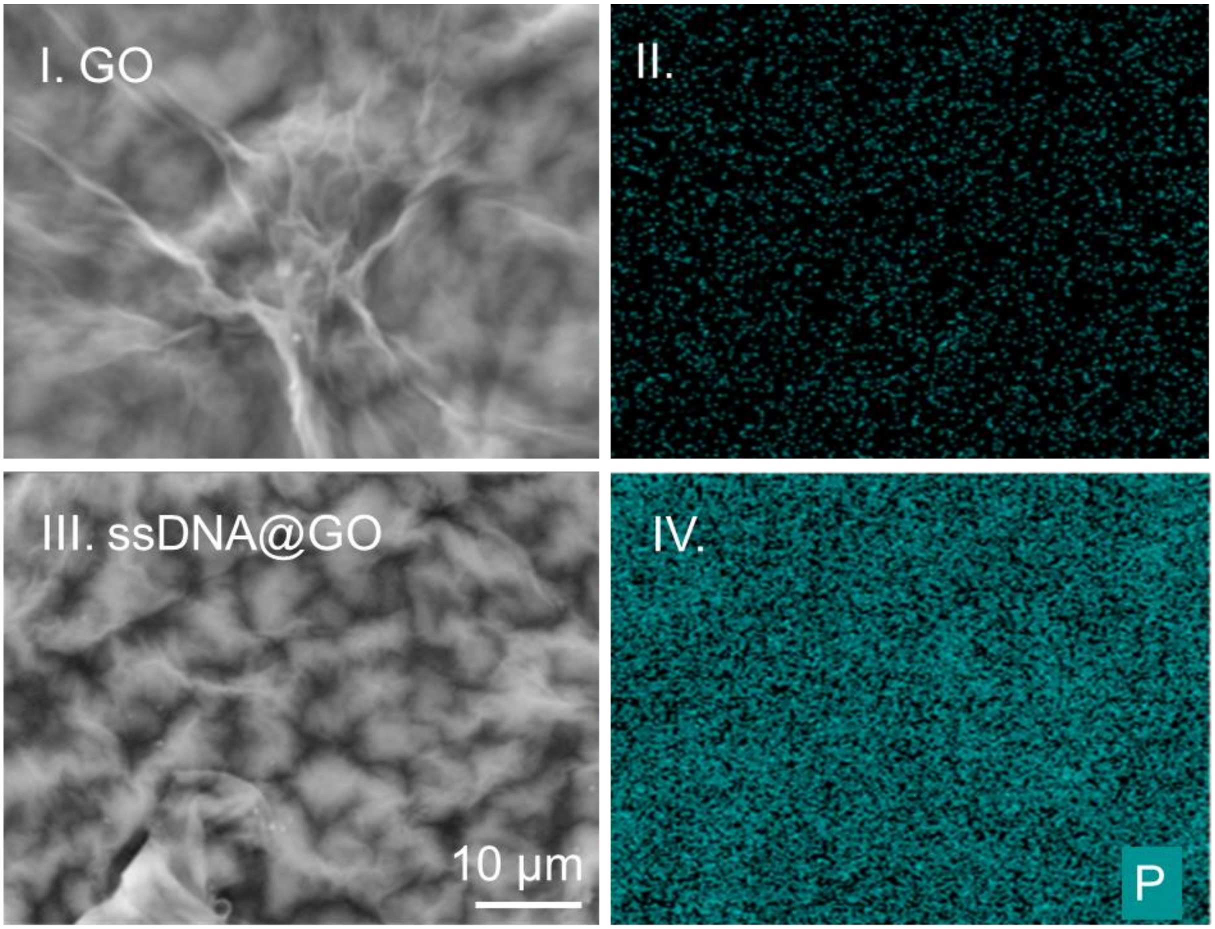

Fig. S23. Energy dispersive X-ray mapping of the P elements from GO membrane (I & II) and ssDNA@GO membrane (III &IV). Scale bar: 10 μm.

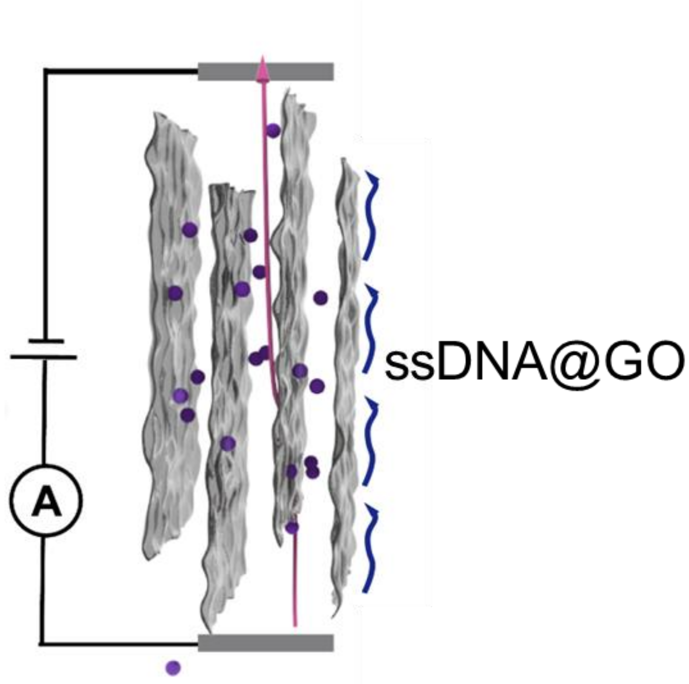

Fig. S24. Illustration for ssDNA@GO membrane.

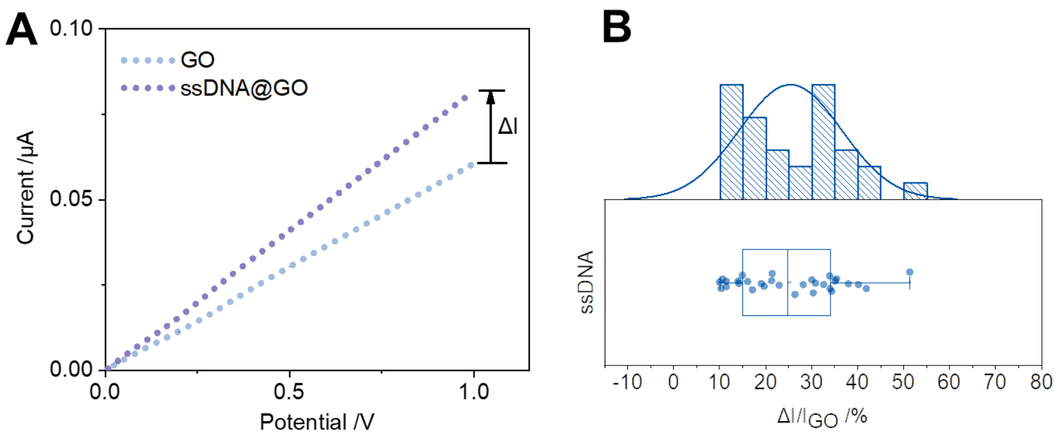

Fig. S25. The ionic currents increase following the modification of ssDNA. (**A**) Typical *I−V* curves of the GO and ssDNA@GO membrane. Compared to GO, the currents of ssDNA@GO membrane increase. The signal of ΔI is defined as the variation in the current at +1.0 V before and after the assembly of ssDNA@GO ($\Delta I = I_{ssDNA@GO} - I_{GO}$). (**B**) The ion current increase ratio ($\Delta I/I_{GO} \times 100\%$ at +1.0 V) after the assembly ssDNA. N = 30 repeats.

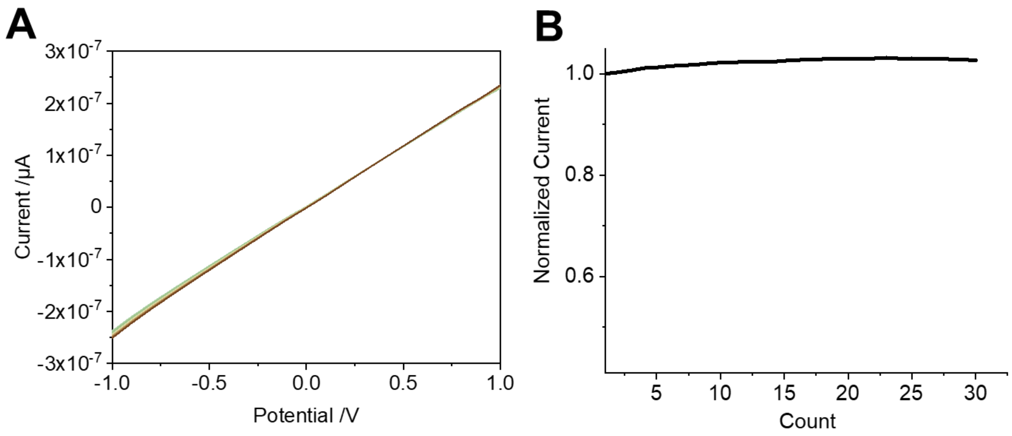

Fig. S26. The long-term stability of ssDNA@PDA-GO membrane. (**A**) *I*−*V* curves from the 1st scan to 30th scan were obtained from ssDNA@GO membrane. (**B**) The normalized current at +1.0 V were obtained from *I*-*V* curves.

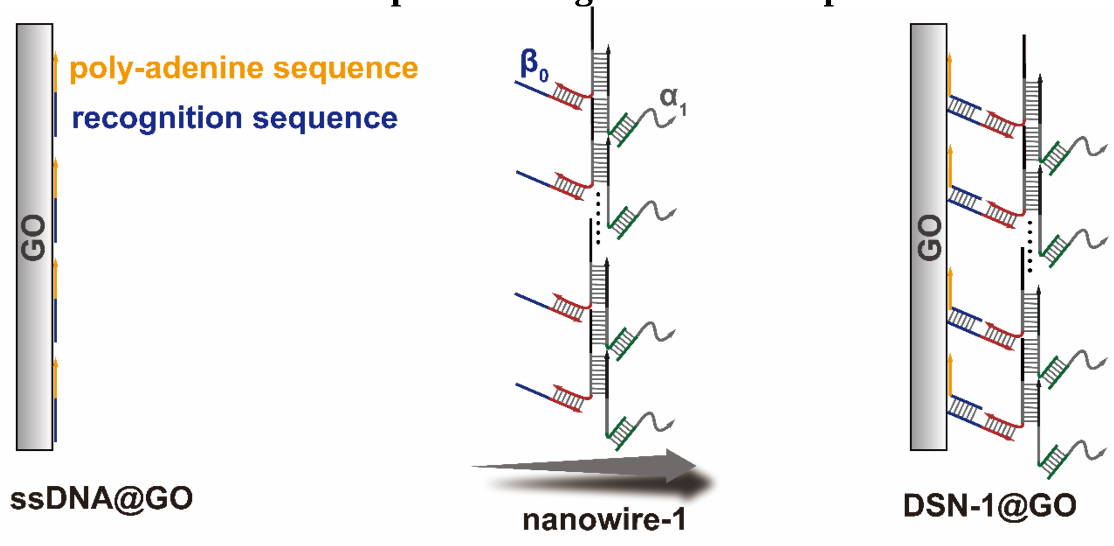

Fig. S27. Sketch illustrating how DSN-1@GO membrane were constructed.
The ssDNA could adsorb onto the surface of GO membrane to fabricate the ssDNA@GO membrane through hydrophobic and π–π stacking interaction. The poly-adenine sequence serves as anchoring block between DNA and GO due to the high binding affinity of poly-adenine to GO. The recognition sequence on ssDNA exhibited the ability to form hybrids with β_0 in nanowire-1, facilitating the DSN-1@GO membrane.

DSN-1 @GO	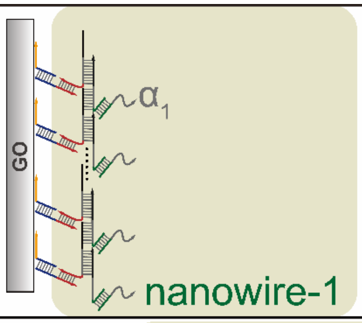
DSN-2 @GO	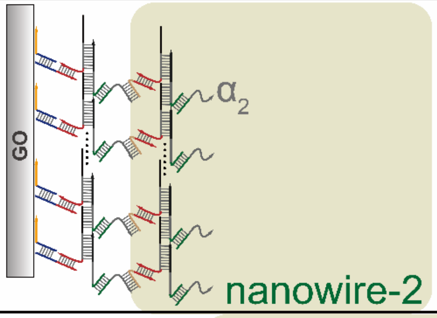
DSN-3 @GO	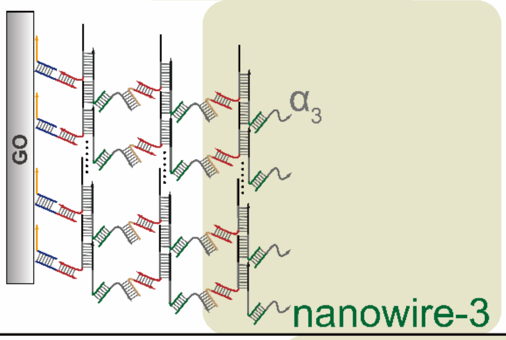
DSN-4 @GO	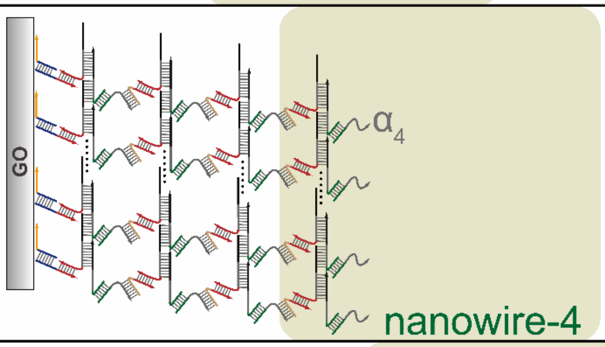
DSN-5 @GO	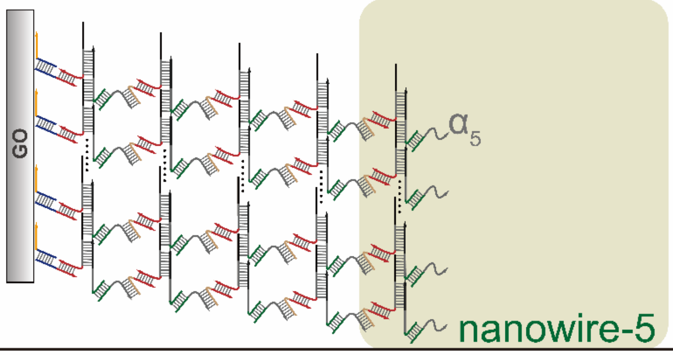

Fig. S28. The 2D schematic diagram of DSN-1, DSN-2, DSN-3, DSN-4, and DSN-5@GO membrane.

The facilitation of DSN-n@GO was achieved via a DNA layer-by-layer self-assembly method. DSN-n@GO membrane including DSN-1@GO assembled by ssDNA and one layer multibranched DNA nanowire (GO, ssDNA, nanowire-1); DSN-2@GO assembled by ssDNA and two layers multibranched DNA nanowires (GO, ssDNA, nanowire-1, nanowire-2); DSN-3@GO assembled by ssDNA and three layers multibranched DNA nanowires (GO, ssDNA, nanowire-1, nanowire-2, nanowire-3); DSN-4@GO assembled by ssDNA and four layers multibranched DNA nanowires (GO, ssDNA, nanowire-1, nanowire-2, nanowire-3, nanowire-4); DSN-5@GO assembled by ssDNA and five layers multibranched DNA nanowires (GO, ssDNA, nanowire-1, nanowire-2, nanowire-3, nanowire-4, nanowire-5).

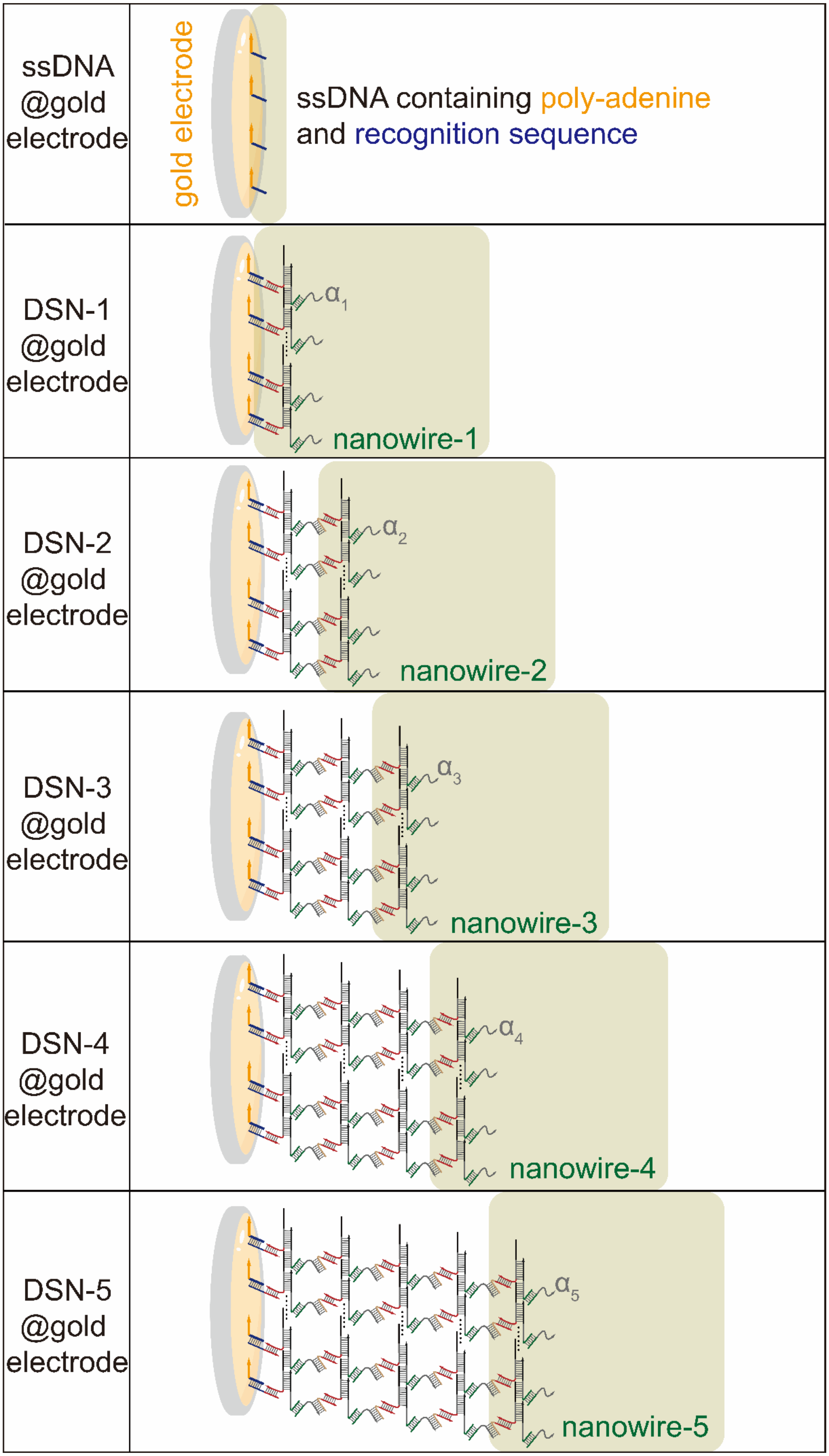
ssDNA @gold electrode
gold electrode
ssDNA containing poly-adenine and recognition sequence
DSN-1 @gold electrode
α1
nanowire-1
DSN-2 @gold electrode
α2
nanowire-2
DSN-3 @gold electrode
α3
nanowire-3
DSN-4 @gold electrode
α4
nanowire-4
DSN-5 @gold electrode
α5
nanowire-5

Fig. S29. The 2D schematic diagram of DSN-1, DSN-2, DSN-3, DSN-4, and DSN-5 on gold electrode for EIS test.

The assembly of DSN on gold electrode follows a similar process as on the GO membrane. As ploy-adenine sequence has a higher affinity to gold than the rest of the DNA, the use of ssDNA containing poly-adenine anchoring sequence was performed. Similarly, the recognition sequence on ssDNA exhibited the ability to form hybrids with β_0 in nanowire-1, facilitating the DSN-1@gold electrode structure. Then DNA layer by-layer self-assembly method was employed to construct DSN-n@gold electrode structure, including DSN-1@gold electrode assembled by ssDNA and one layer multibranched DNA nanowire (gold electrode, ssDNA, nanowire-1); DSN-2@gold electrode assembled by ssDNA and two layers multibranched DNA nanowires (gold electrode, ssDNA, nanowire-1, nanowire-2); DSN-3@gold electrode assembled by ssDNA and three layers multibranched DNA nanowires (gold electrode, ssDNA, nanowire-1, nanowire-2, nanowire-3); DSN-4@gold electrode assembled by ssDNA and four layers multibranched DNA nanowires (gold electrode, ssDNA, nanowire-1, nanowire-2, nanowire-3, nanowire-4); DSN-5@gold electrode assembled by ssDNA and five layers multibranched DNA nanowires (gold electrode, ssDNA, nanowire-1, nanowire-2, nanowire-3, nanowire-4, nanowire-5).

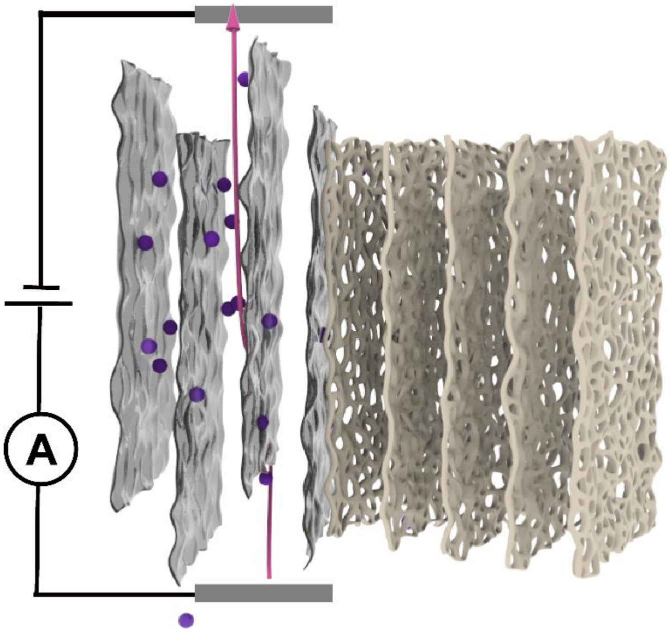

Fig. S30. Illustration for studying ion transport behavior of DSN-n@GO membrane.

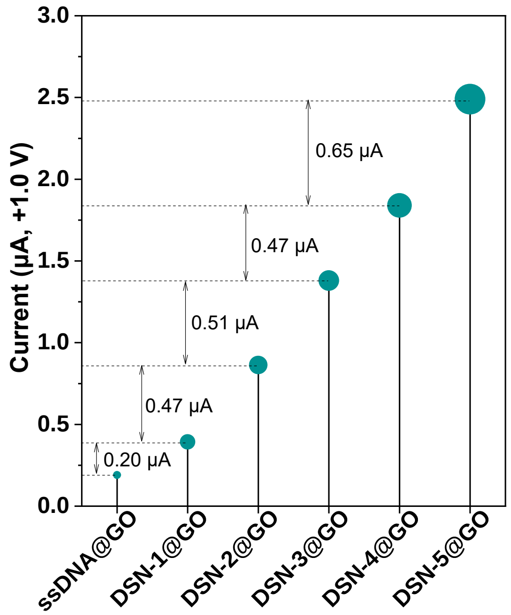

Fig. S31. Summary of ion current changes of bionic membrane system at +1.0 V along with the DNA layer-by-layer self-assembly.

Prior to the self-assembly process, despite the positive charge of PDA molecules, the GO membrane still retained a negative charge. As the layer-by-layer self-assembly went on, the surface negative charge gradually increased, resulting in the current at +1.0 V increased. The ion flux obtained from the DSN-5@GO membrane is 5.71×10^{20} s^{-1} V^{-1} m^{-2}.

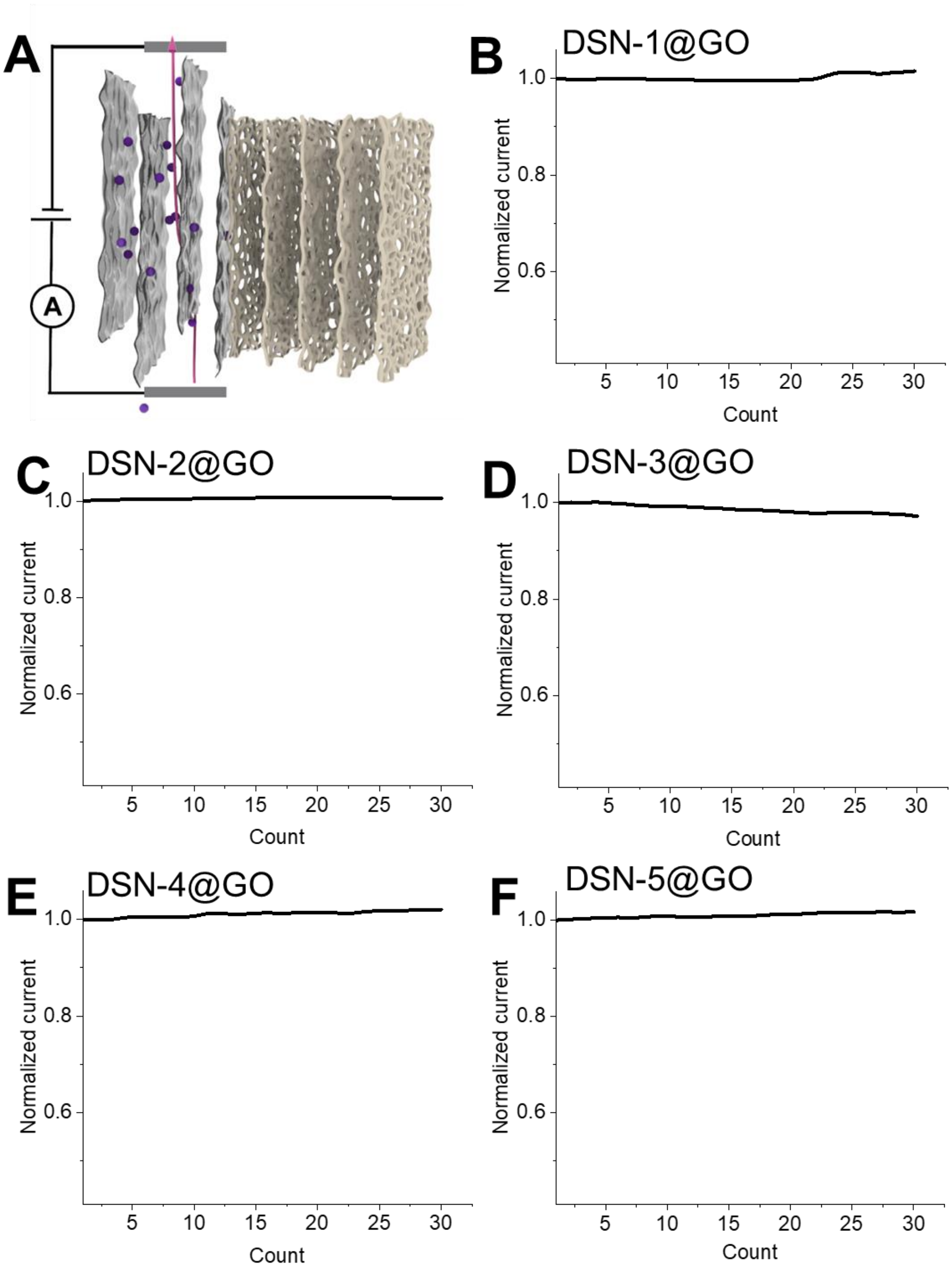

Fig. S32. The stability of DSN-n@GO membrane was investigated by *I-V* tests with 30 continuous repeats. (**A**) Scheme of the of DSN@GO membrane used for tests. The stability of (**B**)DSN-1, (**C**) DSN-2, (**D**) DSN-3, (**E**) DSN-4 and (**F**) DSN-5@GO membrane were investigated by the normalized ion current. The normalized ion signals at +1.0 V were obtained from the corresponding *I-V* curves.

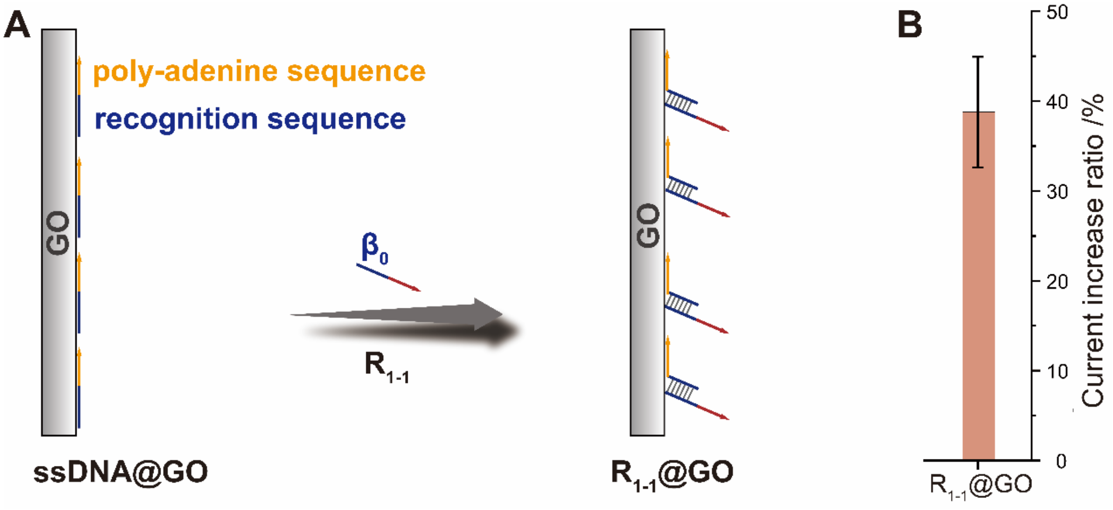

Fig. S33. Design and characterization the role of single-strand DNA on regulating ion transport. (**A**) The two-dimensional (2D) schematic diagram of construction of R_{1-1}@GO membrane. (**B**) The ion current increase ratio $((I - I_0)/I_0 \times 100\%$ at +1.0 V) after the assembly of R_{1-1}. Data represent mean ± SD (N = 3). The presence of the R_{1-1} containing β_0 sequence resulted in a minor change in ion current (~39%), attributable to the lower charge density inherent in the single-strand DNA.

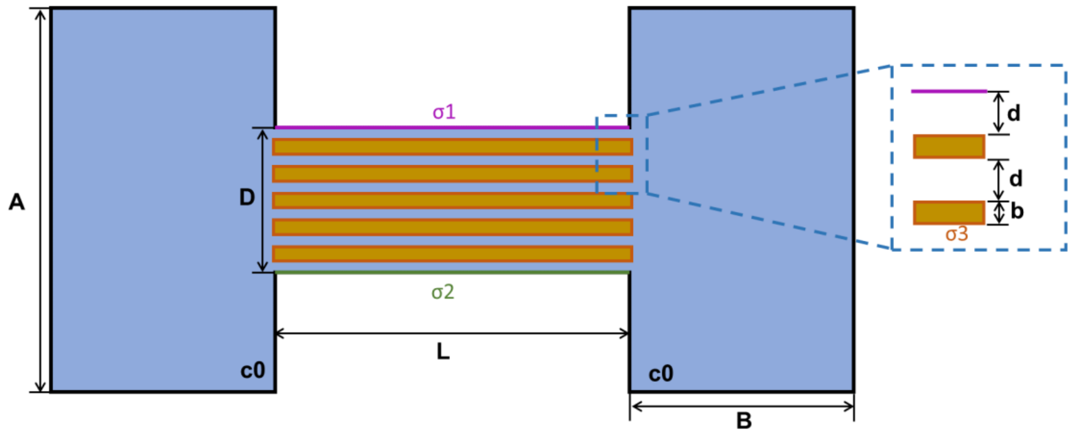

Fig. S34. Schematic illustration of comsol model.
2D planar model was employed to calculate the ion transport in DSN-n@GO membrane. In the 2D model, the calculated domain encompasses two reservoirs linked by a nanochannel measuring 16 nm in width and 100 nm in length, and the nanochannel contains five vertically arranged lamellas. The interlayer spacing of the arranged lamellas is 1 nm, so that the computational accuracy of the continuum model can be ensured. The reservoir dimensions were 2 μm × 4 μm, adequately sized to ensure precise calculation outcomes. The electrolyte used is 10 μM KCl. σ1 represents the surface charge density after DSN-n modification, σ2 denotes the surface charge density of the GO membrane, and σ3 signifies the surface charge density of the GO nanosheets. To ensure convergence of the formula and facilitate computation, all surface charge densities used in the calculation are reduced to one-tenth of the measured data. Detailed settings of model parameters can be found in Table S3.

DSN content regulates membrane compositions

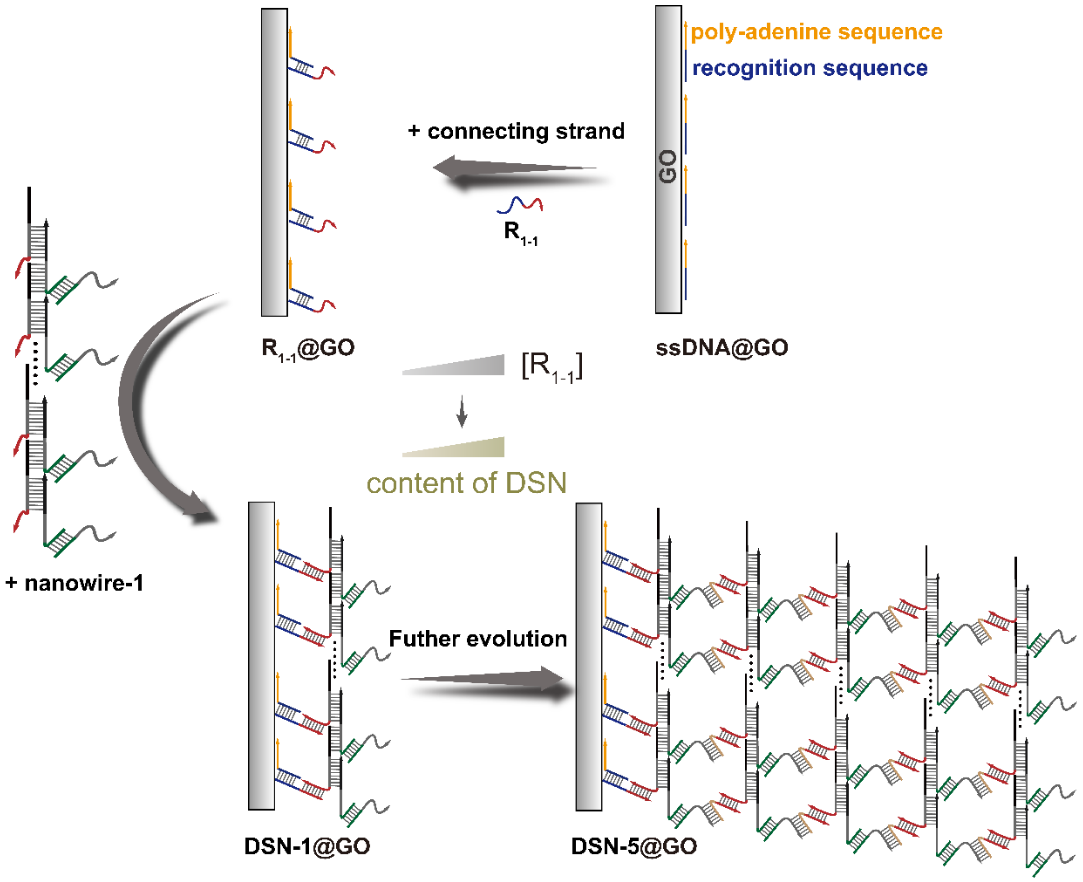

Fig. S35. Illustration of modulation of content of DSN via controlling amount of connecting strand.

The R_{1-1} sequence connected ssDNA@GO membrane with DSN nanostructures. The construction of two kinds of DSN-n@GO membrane, DSN-1@GO and DSN-5@GO membranes, follows the layer-by-layer self-assembly progress.

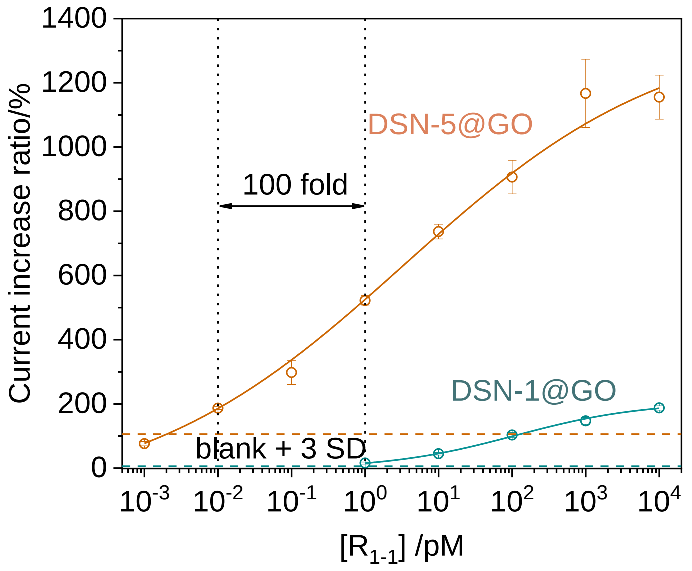

Fig. S36. Current increase ratio measured in DSN-1@GO and DSN-5@GO membrane upon the treatment of different concentrations of connecting strand.

The black dashed lines depict the content modulation limits of connecting strand based on the mean values of the blank sample plus three times the standard deviation (SD). Data represent mean ± SD (N = 3).

Reversible regulation of membrane composition

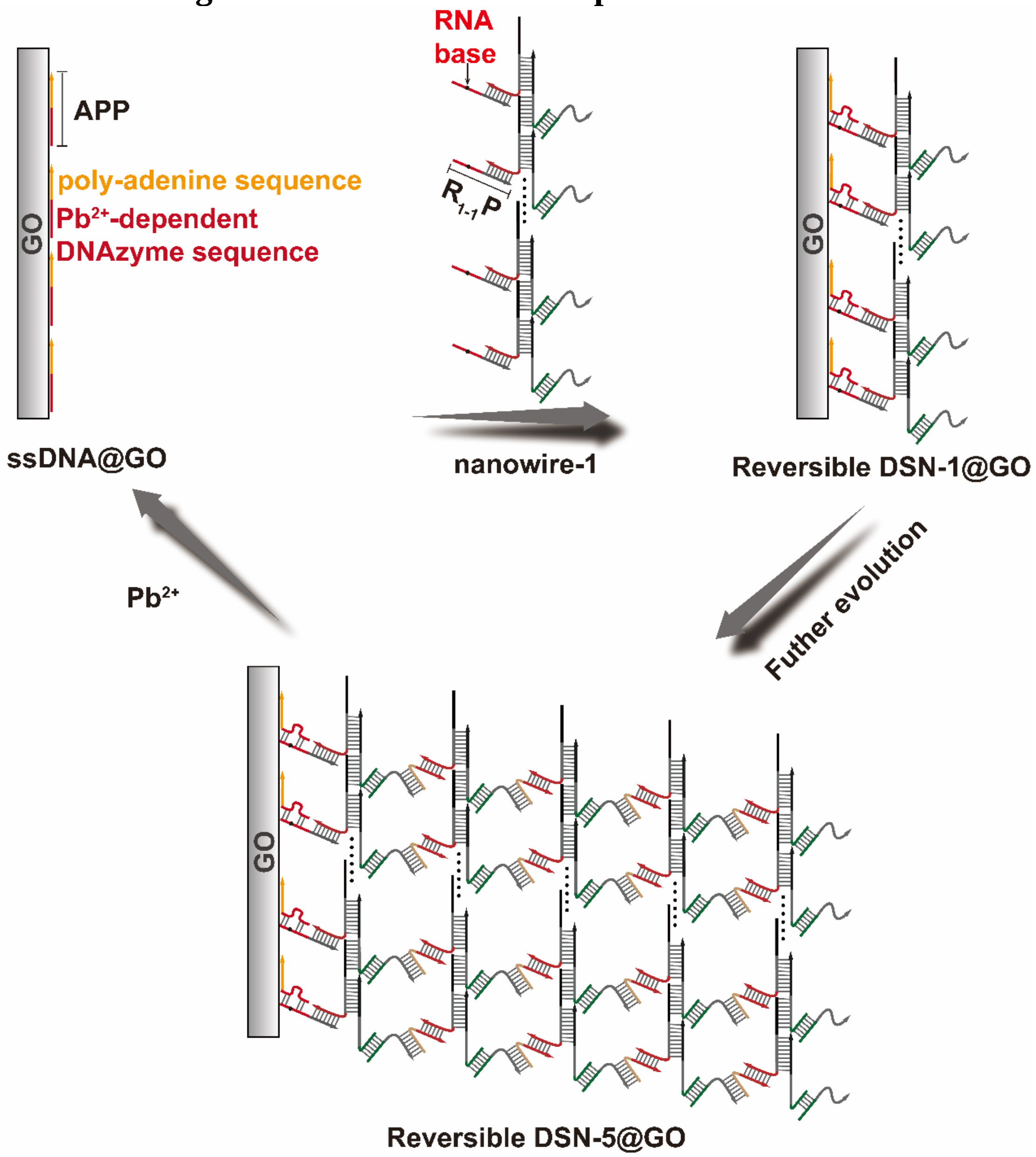

Fig. S37. Illustration of reverse regulation of DSN-5@GO membrane composition.

The single-stranded DNA (APP) integrates a Pb^{2+}-dependent DNAzyme sequence, capable of hybridizing with the DNA-RNA chimeric substrate sequence ($R_{1-1}P$) in nanowire-1. Upon addition of lead ions, cleavage of $R_{1-1}P$ occurs, resulting in the release of DSN-5 from the GO surface.

Table S1. DNA sequences used in this study.

Name	Sequence (5'-3')
S_1	CACATGCAAGAGGTCAAGGCGTCAATTACATTCGTCGACGAGACAG
S_2	GGTGGTGGTGGTTGAAGACCTCTTGCATGTGTGTAATTGACGCCTT
R_{1-1}	AGTATTGCGGAGGAA GGTAAAAAAA CTGTCTCGTCGACGA
R_{2-1}	TCAACCACCACCACC AAAAAAAAAA GAGAGAGAATAAGGG
R_{1-2}	CCCTTATTCTCTCTC AAAAAAAAAA CTGTCTCGTCGACGA
R_{2-2}	TCAACCACCACCACC AAAAAAAAAA AGCACAAGACGACAC
R_{1-3}	GTGTCGTCTTGTGCT AAAAAAAAAA CTGTCTCGTCGACGA
R_{2-3}	TCAACCACCACCACC AAAAAAAAAA GTGGAGTGGAAGTTG
R_{1-4}	CAACTTCCACTCCAC AAAAAAAAAA CTGTCTCGTCGACGA
R_{2-4}	TCAACCACCACCACC AAAAAAAAAA AGATGCACAACAGCA
R_{1-5}	TGCTGTTGTGCATCT AAAAAAAAAA CTGTCTCGTCGACGA
R_{2-5}	TCAACCACCACCACC AAAAAAAAAA CAGCCATTCCGGATG
ssDNA	TTCCTCCGCAATACT AAAAAAAAAAAAAAAAAAAAAAAAAAAAAA
APP	TGAGTGATAAAGCTGGCCGAGCCTCTTCTCTACTTT AAAAAAAAAAAAAAAAAAAAAAAAAAAAAA

$R_{1-1}P$	GTAGAGAAGGAAGGrATATCACTCA GGTAAAAAAACTGTCTCGTCGACGA
1-Mut	AGTAATGCGGAGGAAGGTAAAAAAACTGTCTCGTCGACGA
2-Mut	AGTAACGCGGAGGAAGGTAAAAAAACTGTCTCGTCGACGA
FAM-S_1	FAM-CACATGCAAGAGGTCAAGGCGTCAATTACATTCGTCGACGAGACAG
CY5-ssDNA	CY5-TTCCTCCGCAATACT AAAAAAAAAAAAAAAAAAAAAAAAAAAAAA
Biotin-S_1	Biotin-CACATGCAAGAGGTCAAGGCGTCAATTACATTCGTCGACGAGACAG

Table S2. The zeta potential of GO membrane.

pH	ζ (mV)
6.98	-46.89
6.95	-51.59
6.95	-47.56
6.96	-49.54

Table S3. The parameters of calculated models.

Parameter	Description	Description
A	Length of reservoir	4 μm
B	Width of reservoir	2 μm
D	Diameter of nanochannel	16 nm
L	Length of nanochannel	100 nm
σ1	Surface charge density of top surface	-0.00004, -0.00906, -0.01396, -0.02018, -0.02453, -0.03306 C m^{-2}
σ2	Surface charge density of bottom surface	-0.00004 C m^{-2}
σ3	Surface charge density of nanosheets	-0.00002 C m^{-2}
b	Width of nanosheet	2 nm
d	Interlayer spacing	1 nm
Dp	Diffusion coefficient of K^+	1.957×10^{-5} cm^2 s^{-1}
Dn	Diffusion coefficient of Cl^-	2.032×10^{-5} cm^2 s^{-1}
C0	Electrolyte concentration	10 μM
